# A Genome-Wide Survey of Transgenerational Genetic Effects in Autism

**DOI:** 10.1371/journal.pone.0076978

**Published:** 2013-10-24

**Authors:** Kathryn M. Tsang, Lisa A. Croen, Anthony R. Torres, Martin Kharrazi, Gerald N. Delorenze, Gayle C. Windham, Cathleen K. Yoshida, Ousseny Zerbo, Lauren A. Weiss

**Affiliations:** 1 Department of Psychiatry and Institute for Human Genetics, University of California San Francisco, San Francisco, California, United States of America; 2 Division of Research, Kaiser Permanente Northern California, Oakland, California, United States of America; 3 Center for Persons with Disabilities, Utah State University, Logan, Utah, United States of America; 4 Genetic Disease Screening Program, California Department of Health Services, Richmond, California, United States of America; 5 Division of Environmental and Occupational Disease Control, California Department of Health Services, Richmond, California, United States of America; Emory University School Of Medicine, United States of America

## Abstract

Effects of parental genotype or parent-offspring genetic interaction are well established in model organisms for a variety of traits. However, these transgenerational genetic models are rarely studied in humans. We have utilized an autism case-control study with 735 mother-child pairs to perform genome-wide screening for maternal genetic effects and maternal-offspring genetic interaction. We used simple models of single locus parent-child interaction and identified suggestive results (*P*<10^−4^) that cannot be explained by main effects, but no genome-wide significant signals. Some of these maternal and maternal-child associations were in or adjacent to autism candidate genes including: *PCDH9, FOXP1, GABRB3, NRXN1, RELN, MACROD2, FHIT, RORA, CNTN4, CNTNAP2, FAM135B, LAMA1, NFIA, NLGN4X, RAPGEF4*, and *SDK1*. We attempted validation of potential autism association under maternal-specific models using maternal-paternal comparison in family-based GWAS datasets. Our results suggest that further study of parental genetic effects and parent-child interaction in autism is warranted.

## Introduction

Autism is a heterogeneous neurodevelopmental disorder defined by deficits in language and social behavior as well as patterns of repetitive behaviors. Twin, sibling, and broader autism phenotype research has demonstrated the high heritability of autism spectrum disorders as well as autism-related traits [1], [2], [3], [4]. While family-based studies suggest that the inheritance of autism is complex, the occurrence of autism or autistic traits in individuals with diseases of known genetic etiology (such as Fragile X, Rett or Timothy syndrome) suggests a simpler genetic basis for autism in around 5% of autism cases [5]. The advent of more frequent and higher resolution clinical cytogenetic testing has also shown that genetic copy number variation or other aberrations can be found in 10–20% of individuals with autism, further implicating a highly penetrant genetic basis in some cases not associated with a known genetic disorder [5]. Several genome-wide association studies of common single nucleotide polymorphisms (SNPs) have been performed, but the most significant results from these studies, much like other common complex diseases, show modest effect size [6], [7], [8]. Thus, the genetic etiology of the majority of autism cases remains elusive.

Genetic studies of autism to date have been focused on genetic risk carried by the affected proband. The possibility that parental genotypes influence risk for autism, separately or in conjunction with the genotype of the proband, has yet to be investigated. There are two main types of effects that we consider here: parental main effects, where the genes in one generation have an effect on the phenotype in the next generation independently of inheritance, and transgenerational epistasis, where a combination of genes in the parent and the offspring lead to a phenotype. These effects deviate from classical Mendelian models and have not been widely studied. However, there are several examples in model organisms that demonstrate their importance [9], [10]. These effects are not detectable by the analyses used in most genetic studies, although they would cause clustering in families and patterns of inheritance that may appear indistinguishable from complex heritable main proband effects.

There are several models through which transgenerational effects could contribute to autism risk. The first model is in relation to maternal main effects; specifically, this model postulates that genetic variation in the mother alters the fetal environment and impacts early neurodevelopment. One well-documented example of this type of maternal effect can be seen in neural tube defects (NTDs). It has been shown that low folate and vitamin B_12_ in the mother increase the risk of neural tube defects. Additionally, several groups have demonstrated that maternal genetic polymorphisms in *MTHFR*, which has a role in folate metabolism, and *TCN2*, which has a role in vitamin B_12_ transport, have an effect on the risk of NTDs [11], [12].

The second model is that of maternal-fetal genetic interaction. One example of this might be genetic incompatibility. It is long-recognized that maternal-fetal cellular exchange occurs during pregnancy and birth and can have consequences for the fetus/neonate or the mother. Further, research has shown that fetal cells exist and survive long-term in the maternal system in a phenomenon known as microchimerism [13]. Thus, if the fetus expresses an allele the mother does not, resulting in a foreign antigen, a damaging maternal immune response could be activated. Conversely, maternal cells can integrate into the fetus during gestation, and survive postnatally [14]. There is also evidence that microchimeric cells may cross the blood-brain barrier [15]. As a result, if the mother expresses an allele her offspring does not, the maternal cells could present an antigen foreign to the offspring. Finally, genetic similarity at specific compatibility loci could allow for integration of foreign cells which are ultimately disruptive [16]. Several approaches for analyzing maternal-fetal incompatibility and transgenerational epistasis have previously been described [17], [18].

A well-understood example of transgenerational epistasis leading to a damaging maternal immune response in humans is Rh disease. Rh disease occurs when the fetus inherits an Rh+ allele from the father and the mother is Rh negative, causing a maternal immune response against the foreign Rh antigen expressed by the fetal blood cells. Some evidence has pointed to Rh incompatibility as a risk factor for neuropsychiatric disorders, such as schizophrenia [19]. In support of this model in autism, several studies have found maternal antibodies to fetal brain antigens in serum from mothers of autistic children [20], [21]. This mechanism has also been observed in animal models. Martin *et al*. found that rhesus macaques infused mid-gestation with serum from human mothers of children with autism went on to have offspring with autism-like phenotypes [22].

In this study, we have used novel analyses to test specific models of maternal genetic effects in autism. We report the results of a case-control study using mother-child pairs to test novel models to investigate proband genetic main effects, maternal genetic main effects, and maternal-fetal transgenerational epistasis in autism.

## Methods

### Ethics Statement

The committee for the Protection of Human Subjects (CPHS), California Health and Human Services Agency, an institutional review board that operates in compliance with the Common Rule under the authority of a Federalwide Assurance, reviewed the project and granted a waiver of HIPAA authorization. A designated member of the Kaiser Permanente Northern California (KPNC) Institutional Review Board (IRB) approved this study, including waiving the requirement for informed consent and waiving the requirement that Privacy Rule authorization be obtained from study participants. The Utah State University IRB granted this project (Protocol #1548) an Exemption because the samples were pre-existing and no identifying information accompanied the samples handled at the USU laboratory. The UCSF Committee on Human Research considers this to be non-human subject research, meeting the criteria that 1) The coded private information and specimens were not collected specifically for the current research project and 2) UCSF PI and holder of the key have an agreement prohibiting the release of the key (EMA) or there are IRB-approved written policies for the repository or data management that prohibit the release of the key (replication data).

### Primary study samples

Our samples are part of the Early Markers for Autism (EMA) study, which has been previously described [21], [23]. Briefly, EMA is a large, population-based, nested case-control study of autism that utilizes archived prenatal (maternal blood cell pellet) and newborn (neonatal blood spot) specimens (banked at −20°C) from mother-baby pairs. The study population derives from women in Orange, San Diego and Imperial Counties, California who were pregnant in 2000–2003 and who enrolled in the State's Prenatal Expanded Alphafetoprotein Screening Program. These participants were self-reported to be: 35% White, 42% Hispanic, 18% Asian, 3% African, 2% Other. They are well-matched between cases and controls, but to avoid population stratification we have employed empirical clustering based on genetic data (see Genetic Analysis below). Three groups of children born to these women were identified: children with autism spectrum disorder (ASD), children with other developmental delay (DD) but not ASD, and general population controls (GP). Children with ASD or DD were ascertained from client records of the Regional Center of Orange County (RCOC), and San Diego Regional Center, two of the 21 Regional Centers operated by the California Department of Developmental Services (DDS) to coordinate services for persons with autism, developmental delay, and other developmental disabilities. Clients receiving DDS services for autistic disorder or for other DDS eligible conditions with suspected ASD were ascertained as possible ASD cases for this study. GP controls were randomly sampled from the birth certificate files after past or current DDS/RC clients had been excluded, and matched to ASD cases by sex, birth month and birth year.

ASD and DD diagnoses were verified by abstraction and expert review of Regional Center medical records following a protocol initially developed by the Metropolitan Atlanta Developmental Disabilities Surveillance Program [24]. Maternal mid-pregnancy (15–20 weeks gestation) venous blood specimens and newborn bloodspots were retrieved from the prenatal and newborn screening specimen archives maintained by the Genetic Disease Screening Program, California Department of Public Health. All study procedures were approved by the institutional review boards of the California Health and Human Services Agency, Kaiser Permanente Northern California, and UCSF.

### DNA Extraction

Specimens to be tested were coded with a study identification number. For maternal samples, blood cell pellets collected in 4ml serum separator tubes were shipped on dry ice to the biomedical laboratory at Utah State University for processing. The cell pellets were broken up with pipette tips and about 400 ul from each sample was removed and placed into test tubes. The QIAGEN QIAamp 96 DNA Blood Kit was used following the manufacturer's protocol.

For newborn samples, one dried bloodspot (about 14 mm in diameter) collected on filter paper was entirely punched with a 3.2 mm paper punch. Since there is little DNA in a 3.2 mm paper punch, 15 to 18 punches were used for DNA extraction. Again, the QIAGEN QIAamp 96 DNA Blood Kit was used following the manufacturer's protocol. Three of the 3.2 mm punches were used per well.

The Invitrogen Quant-iT DNA Assay Kit was used to measure the DNA concentration and the spectrophotometer absorbance was measured at 260/280 and 260/230 on 3 ul of sample to determine DNA purity (Bio-Tek Synergy H instrument using the Take3 plate).

### Genotyping

All maternal and neonatal samples were genotyped using the Affymetrix Axiom EUR array by the Genomics Core Facility (GCF) at UCSF, using standard protocols. The Axiom EUR array assays approximately 675,000 SNPs across the genome, and is optimized for genome-wide, gene-based, and candidate-SNP coverage [25]. Genotype calling was carried out using Affymetrix Power-Tools in accordance with the Axiom Genotyping Solution Data Analysis Guide provided by Affymetrix [26], [27].

Genotype calling and quality control were carried out separately for the neonatal bloodspot-derived DNA and maternal blood pellet DNA samples. For samples to be included in analysis, we required a genotype call rate >97%. We checked genetic relationships using PLINK's ‘–genome’ module to calculate pair-wise genetic relatedness between all individuals in our sample [28]. We used this information to correct any sample switches that were resolvable. Any additional pairs that were genetically unrelated were considered to be misidentified and excluded. We also calculated the F inbreeding coefficient for each sample and average pi-hat using PLINK [28]. Samples that were found to be outliers with F inbreeding coefficient significantly less than zero and high average pi-hat were considered contaminated.

We performed Axiom genotyping on DNA from 1,706 EMA samples. Among our neonatal bloodspot samples (N = 852), 3 (0.03%) were excluded due to failed genotyping, 36 (4.2%) were excluded due to low genotype call rate, 13 (1.5%) were excluded due to apparent contamination, 26 (3.1%) were excluded due to sample misidentification, and 10 (1.2%) were removed due to unresolved affection status resulting in 88 (10%) total excluded samples. Among our maternal blood pellet samples (N = 854), 14 (1.6%) were excluded due to low genotype call rate, 18 (2.1%) were excluded due to apparent contamination, 22 (2.6%) were excluded due to sample misidentification, and 10 (1.2%) were removed due to their child's unresolved diagnosis, resulting in 64 (7.5%) total excluded samples. The higher rate of samples excluded due to low call rate among the neonates (4.2%) compared to the mothers (1.5%) is consistent with the quality of DNA extracted from the bloodspot samples being on average lower than the quality available from the blood pellet samples. After quality control, we were left with 764 offspring samples (385 cases, 379 controls) and 790 maternal samples (390 cases, 400 controls). These samples comprise 735 complete maternal-offspring pairs (366 cases, 369 controls).

We filtered out markers with genotype call rate <97%, Fisher's Linear Discriminant (FLD) score <3.6, and Heterozygous cluster strength offset (HetSO) value <−0.1 as outlined in the Axiom Advanced Analysis Workflow. After these steps were carried out separately in the maternal and neonatal sample sets, the samples were combined and markers that were excluded in either set were subsequently excluded from transgenerational analysis. After combining the maternal and neonatal samples, we tabulated Mendelian errors of inheritance in all of our complete maternal-baby pairs and additionally excluded markers with >10 of these errors. We also excluded markers that violated Hardy Weinberg Equilibrium (HWE) in control mothers, as judged by a *P*-value <10^−10^ using the Hardy-Weinberg exact test statistic [29]. These quality control steps were carried out using PLINK [28]. Out of the 674,557 markers assayed by the Axiom EUR platform, 653,758 (97.0%) passed QC metrics in the neonatal samples and 659,993 (97.8%) passed QC metrics in the maternal samples. This is consistent with sample quality differences expected between neonatal blood spots and maternal blood pellets. 647,227 markers (96.0%) passing in both maternal and neonatal samples were included in our combined analysis.

### Replication Datasets

Replication data are comprised of five datasets that have been used in previous and ongoing GWAS of autism. The first dataset is comprised of samples genotyped by the Autism Genome Project (AGP) and includes 4,074 samples; these samples were genotyped using the Illumina Human 1M-single Infinium BeadChip array [8]. The second dataset includes samples from the Autism Genetic Resource Exchange (AGRE) and the National Institute for Mental Health (NIMH, collections of DNA from multiplex families with ASD by the NIMH Autism Genetics Initiative); it includes 3,717 samples genotyped on the Affymetrix 500 k and Affymetrix 5.0 platforms [6]. The third dataset also consists of AGRE multiplex families and includes 4,327 samples. These samples were genotyped on the InfiniumII Illumina 550 k Bead Chip platform [7]. The fourth and fifth datasets contain samples collected by the Simons Simplex Collection (SSC) and include a total of 4,348 samples; 3,013 of these samples were genotyped on the Illumina 1M Duo platform, and 1,335 were genotyped on the Illumina 1 M platform [30], [31]. These two groups of samples were considered separately. Overall, we utilized 13,373 individuals (3,314 complete families) for the purposes of replicating the proband main effects, maternal main effects, and transgenerational epistatic effects we observed in the EMA discovery data (Methods S1, Table S1).

### Imputation of Replication Datasets

In order to replicate our results using datasets genotyped on different array platforms, we imputed our replication samples using BEAGLE (Version 3.3.2– http://faculty.washington.edu/browning/beagle/beagle.html) with 1000genomes reference data (http://bochet.gcc.biostat.washington.edu/beagle/1000_Genomes.phase1_release_v3/) [32]. Quality control was applied to each dataset individually before and after imputation. Markers with lower than 90% imputed genotype calls were excluded from all analyses and considered to have failed imputation; markers that had 90% imputed genotype calls or greater in any subset of data were considered in analysis (Methods S2).

### Genetic Analysis

In order to address population stratification in our sample, we employed the Cochran-Mantel-Haenszel (CMH) test for repeated tests of independence to compare the relative proportions of allele frequencies or binary classifiers between cases and controls. In order to stratify pairs for the purposes of this CMH test we clustered samples using PLINK's ‘–cluster’ module [28], and then classified each pair based on the maternal cluster solution for paired tests and based on the proband solution for the main effects case-control test (Methods S3). The only clustering restraint we imposed on the algorithm was cluster number (using the – K option). In order to set this tuning parameter in a non-arbitrary fashion, we chose the value of K which minimized the overall genomic inflation in the dataset, as measured by looking at the results of the CMH tests in both the neonates and the mothers. The number of clusters that turned out to minimize the genomic inflation in both tests was 9 (Figure S1).

We carried out case/control tests separately in the maternal and neonatal samples using PLINK's ‘– cmh’ module [28]. For the probands, this constitutes a case/control proband main effect GWAS for autism. For the mothers, this represents a novel genome-wide examination of maternal genetic main effects in autism, with the ‘affected’ phenotype defined by having a child with autism.

**Figure 1:**
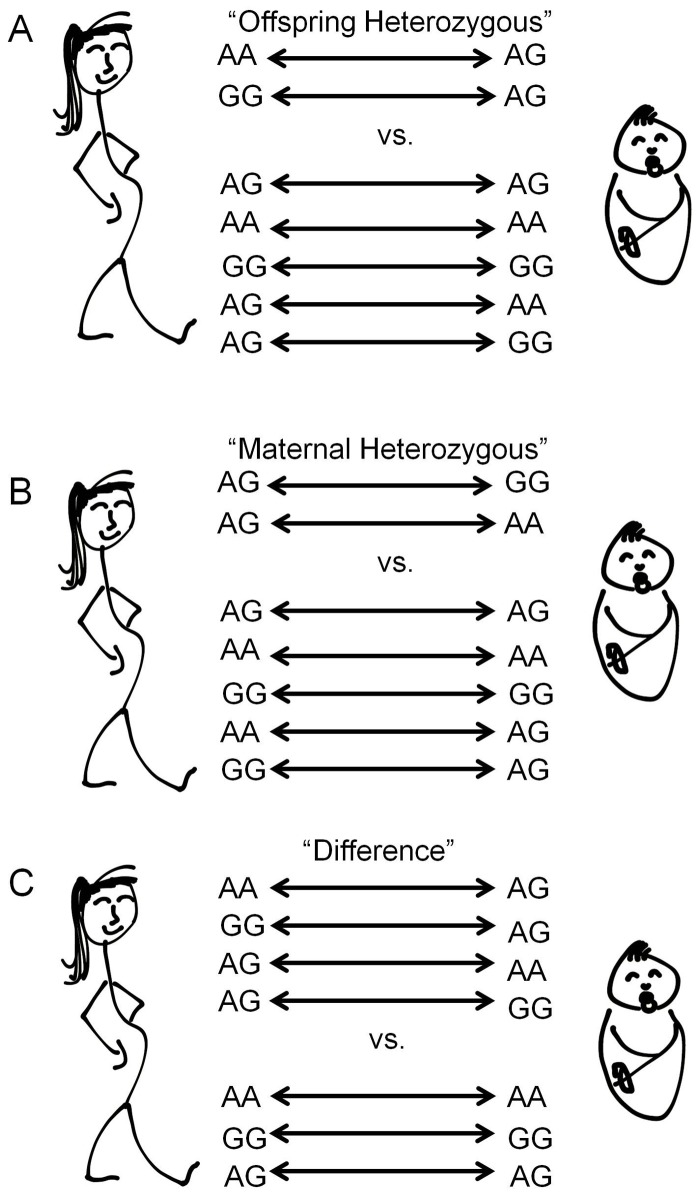
Models of Transgenerational Epistasis. A) “Offspring Heterozygous” model where the offspring has an allele the mother does not vs. pairs where the mother possesses at least one copy of each allele present in the offspring. B) “Maternal Heterozygous” model where the offspring has an allele the mother does not vs. pairs where the mother possesses at least one copy of each allele present in the offspring. C) “Difference” model where the mother and offspring genotypes are identical vs. pairs where they are not identical.

To test the hypotheses of transgenerational epistasis, we created three binary classifiers, as follows: 1) pairs where the offspring has an allele the mother does not vs. pairs where the mother possesses at least one copy of each allele present in the offspring (“Offspring Heterozygous” model), 2) pairs where the mother has an allele the offspring does not vs. pairs where the offspring possesses at least one copy of each allele present in the mother (“ Maternal Heterozygous” model), 3) pairs where the mother and offspring genotypes are identical vs. pairs where they are not identical (“Difference” model) as shown in Figure 1. A CMH test of the proportion of binary classifiers between the case and control pairs for each model was performed.

Using the determined population strata, our genome wide CMH tests in the EMA discovery sample of proband main effects (λ = 1.002), maternal main effects (λ = 1.011), “Offspring-Heterozygous” (λ = 0.991), “Maternal-Heterozygous” (λ = 0.997), and “Difference” transgenerational epistatic effects (λ = 0.998) had no genomic inflation with accompanying q-q plots shown in Figure S2.

In order to demonstrate that maternal effects are not driven by proband main effects, and that transgenerational effects are not driven by a combination of maternal and proband main effects, we employed a multinomial maximum likelihood model (MMLM) as implemented in the EMIM software package (http://www.staff.ncl.ac.uk/richard.howey/emim) and described extensively [18], [33]. By fitting a model that allows for proband and maternal main effects and removing the maternal main effects using a likelihood ratio test (LRT), we can test whether results of the maternal main effects can be explained entirely by proband main effects. Similarly, by fitting a model that allows for maternal and proband main effects as well as transgenerational effects, then removing the transgenerational effects and comparing the model fit, we are able to test whether significant results obtained in the transgenerational epistatic models can be explained entirely by main effects.

To assess fit in our proposed transgenerational effect models, the EMIM package estimates probabilities for *α* to represent the baseline disease risk for a proband, *R_1_* as the risk factor when the proband has one copy of the risk allele and *R_2_ = R_1_^2^* if two copies are present, *S*_1_ as the risk factor when the paired mother possesses a copy of the risk allele and *S_2_ = S_1_^2^* if two copies are present, and an interaction parameter to represent the transgenerational model of interest (e.g. *j_c_* for the Difference model). These parameters are estimated from the pedigree data assuming HWE and random mating. To assess fit in our proposed transgenerational effect models, we define the null model to account for neonatal and maternal genetic effects and our model of interest includes the additional interaction parameter. For example, in our Difference model where a mother is homozygous and the neonate is heterozygous, we would consider *α
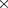
R_1
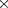
_S_2_* as the null model and contrast it to our model of interest *α
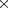
R_1
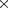
_S_2
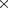
_j_c_* using a LRT:
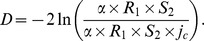



Further methods and parameters are given in Methods S4, Table S2, and Table S3.

### Family-based Replication

In order to replicate the results of case/control tests in the probands, we carried out transmission disequilibrium tests (using PLINK's TDT module) in available family-based autism datasets [28]. We used the same family-based data to replicate the maternal effects, performing a chi-square test of allele frequency in PLINK with the mothers considered “cases” and the fathers considered “controls” [28].

We devised equivalent tests to our transgenerational epistasis models for replication in the available family-based autism datasets. To replicate the “Offspring Heterozygous” model, we considered all affected offspring trios with heterozygous offspring and compared the number of homozygous mothers to homozygous fathers using a chi-square test. To replicate the “Mother Heterozygous” model, we considered all affected offspring trios with homozygous offspring and compared the number of heterozygous mothers to heterozygous fathers. To replicate the “Difference” model, we considered all affected offspring trios and compared the number of mothers vs. fathers with the opposite genotype as the affected proband (Methods S5, Figure S3).

Several datasets genotyped on different platforms and collected by research consortia with disparate recruitment methods were used for replication. Therefore, we carried out random-effects meta-analysis in PLINK to combine the results into one meta-replication statistic specifying *P* – values, odds ratios and standard errors as input for all models in each dataset [28]. For each of the five main tests (two main effect tests and three transgenerational epistasis tests) the replication *P*-value is from the random-effects meta-analysis statistic. In cases where an allelic combination had zero observations in any single dataset, the replication datasets were considered jointly and the merged replication *P*-value is presented.

## Results

### Proband Main Effects

We carried out a CMH test of allele frequency over 653,758 high-quality markers for the 766 neonatal samples. Our strongest associations (*P*<10^−5^) were on chromosome 8p between *CSMD1* and *MCPH1* (rs28374251, *P* = 3.48×10^−6^), 13q in the intron of *SPATA13* (rs7331042, *P* = 4.22×10^−6^), 8q in the intron of *COL22A1* (rs12680005, *P* = 7.01×10^−6^) and14q between *GCH1* and *WDHD1* (rs76271340, *P* = 9.90×10^−6^) (Table 1). All Proband Main Effect results with CMH *P*<10^−4^ are listed in Table S4.

**Table 1:** Top results (*P*<10^−5^) from CMH test of allele frequencies in main effect and transgenerational effect models.

Model	SNP	Location	Nearest Gene(s)	CMH *P-*val	CMH OR	LRT *P*-val	Rep. *P*-val	Rep. OR
Proband ME	rs28374251	intergenic	*CSMD1* (cub-sushi domain containing 1) – blocks complement pathway activation; enriched in the nerve growth cone in rats [55] *MCPH1* (microcephalin 1) – causes autosomal recessive microcephaly; disrupted by CNVs in autism [56], [57]	3.48×10^−6^	0.60	n/a	0.930	1.00
Proband ME	rs7331042	intronic	*SPATA13* (spermatogenesis associated protein 13; aka *ASEF2*, APC-stimulated guanine nucleotide exchange factor 2) – involved in PI3K-mediated cell migration; enriched expression in the amygdala [58], [59]	4.22×10^−6^	1.64	n/a	failed to impute	n/a
Proband ME	rs12680005	intronic	*COL22A1* (collagen, type XXII, alpha 1) – localizes to tissue junctions [60]	7.01×10^−6^	0.48	n/a	failed to impute	n/a
Proband ME	rs76271340	intergenic	*GCH1* (GTP cyclohydrolase 1) – critical for tertahydrobiopterin biosynthesis; positive regulator of nitric oxide synthesis in umbilical vein endothelial cells; mutations in this gene can cause hyperphenylalaninemia and defective neurotransmission due to depletion of dopamine and serotonin [61] *WDHD1 *(WD40 repeat containing-high mobility group box DNA binding protein 1) – RNA-mediated epigenetic control of centromere integrity and genomic stability [62]	9.90×10^−6^	5.05	n/a	failed to impute	n/a
Maternal ME	rs1940153	intronic	*MAML2* (mastermind like 2) – transcriptional co-activator for Notch proteins [41]	2.05×10^−6^	1.68	0.0012	0.542	1.02
Maternal ME	rs9895531	Intronic	*GAS7* (growth arrest-specific 7) – expressed predominantly in mature Purkinje neurons, proposed to play a role in neurite projection and influence temporal lobe size [63], [64]	5.45×10^−6^	1.61	4.79×10^−6^	0.540	0.98
Maternal ME	rs528615	Intergenic	*PRKACB* (cAMP dependent protein kinase catalytic subunit beta) – has several brain-specific isoforms *SAMD13* (sterile alpha motif containing protein) – unknown function	6.19×10^−6^	0.61	9.63×10^−5^	0.548	0.99
Maternal ME	rs2006933	Intergenic	*TMEM97* (transmembrane protein 97) – controls cellular cholesterol level; upregulated in ovarian surface cells treated with progesterone [65] *NLK* (nemo-like kinase) – links the MAP kinase and Wnt signaling pathways	7.81×10^−6^	1.71	3.59×10^−5^	0.099	1.07
Maternal ME	rs8001767	Intergenic	*KLF5* (Kruppel-like transcription factor 5) – KLFs are critical to vertebrate development and influenced by mastermind-like mediated Notch signaling; expressed in placenta *KLF12* (Kruppel-like transcription factor 12) – see above; expressed in neural progenitors [42]	9.95×10^−6^	1.72	8.24×10^−4^	0.218	0.95
Offspring Het	rs1527470	3′ UTR	*SEMA3C* (semaphorin 3C) – secreted axonal growth cone guidance molecule	5.34×10^−6^	2.28	0.0037	0.954^ *^	1.00
Offspring Het	rs280039	Intronic	*ACCN1* (Amiloride-sensitive cation channel 1, neuronal) – member of the epithelial sodium channel superfamily	7.68×10^−6^	2.24	0.0050	0.539	0.93
Offspring Het	rs959246	Intronic	*SLC14A2* (solute carrier family 14 member 2) – urea transporter	9.42×10^−6^	0.42	1.41×10^−4^	0.996	0.99
Maternal Het	rs10816846	Intronic	*PALM2* (paralemmin 2) – plays a role in plasma membrane dynamics [66]	1.44×10^−6^	0.24	1.39×10^−4^	0.874	1.01
Maternal Het	rs2071330	Intronic	*KIAA0430* (limkain b1) – peroxisomal protein that can be autoimmunogenic [45]	9.69×10^−6^	0.46	0.0100	0.232	1.14
Difference	rs28539905	Intergenic	*C4orf37* (chromosome 4 open reading frame 37) – unknown function *UNC5C* (unc-5 homolog C) – netrin receptor involved in axon guidance	1.99×10^−6^	2.57	0.0206	0.153	1.11
	rs7691268	Intergenic		4.12×10^−6^	2.61	0.0453	0.074	1.17
Difference	rs939046	Intergenic	*INSC* (inscuteable homolog) – determines polarization in neuroblast division [67] *SOX6* (sex determining region y box 6) – transcription factor required for normal development of the central nervous system [68]	2.03×10^−6^	0.48	2.46×10^−6^	0.025	1.10
Difference	rs59358210	Intronic	*SLC7A8* (solute carrier family 14 member 2) – urea transporter	7.86×10^−6^	0.41	0.0078	failed to impute	n/a
Difference	rs7171512	intronic	*GABRB3* (gamma-aminobutyric acid A receptor, beta 3) – has been associated with autism and lies within an imprinted region [46]	9.40×10^−6^	2.12	0.0326	0.185	0.82

SNPs with *P*<10^−5^ in the EMA discovery sample are listed. The model type (Model) and SNP identity (SNP) are shown. For each SNP, the closest annotated genes are indicated as well as relative SNP position to those genes (Nearest Gene(s) and Location). *P*-values (CMH *P*-value) and odds ratios (CMH OR) are shown for a Cochran-Mantel-Haenszel test of pair-type counts in case vs. control pairs from the EMA discovery cohort. In order to show that our models under investigation are not driven by proband main effects (in maternal main effect model) or both maternal and proband main effects (in transgenerational effect models), a comparison between multinomial models is shown (LRT *P*-value). Replication datasets were imputed to allow maximum coverage of SNPs across different platforms. Replication was performed on trios; results were then combined across replication datasets using random-effects meta-analysis (Rep. *P*-value, Rep. OR). *Indicates that a merged Rep. *P-*value and OR are presented rather than the meta-analyzed statistic.

### Maternal Main Effects

In order to assess main effects of maternal genotype, we performed a CMH test of allele frequency over 659,993 high-quality markers for the 800 maternal samples. To separate maternal main effects from proband main effects, we carried out a comparison between multinomial maximum likelihood models (MMLMs) including both maternal and proband main effects and only proband main effects using a likelihood ratio test (LRT). We found that in 93% (54/58) of our top results under the maternal main effect test, a model which included both maternal and neonatal main effects showed a significantly better fit than a model including only neonatal main effects (LRT *P*-value <0.05). Our strongest associations (*P*<10^−5^) that also showed evidence for maternal main effect in our MMLM (LRT *P*<0.05) were on chromosome 11q in the intron of *MAML2* (rs1940153, *P* = 2.05×10^−6^, LRT *P* = 0.00122), 17p in the intron of *GAS7* (rs9895531, *P* = 5.45×10^−6^, LRT *P* = 4.79×10^−6^), 1p between *PRKACB* and *SAMD13* (rs528615, *P* = 6.19×10^−6^, LRT *P* = 9.63×10^−5^), 17q between *TMEM97* and *NLK* (rs2006933, *P* = 3.08×10^−6^, LRT *P* = 9.1×10^−4^) and 13q between *KLF5* and *KLF12* (rs8001767, *P* = 9.95×10^−6^, LRT *P* = 8.24×10^−4^) (Table 1). All Maternal Main Effect results with CMH *P*<10^−4^ are listed in Table S5.

### Transgenerational Epistatic Effects

We performed novel tests of transgenerational epistatic effects, including the LRT to assess the fit of these epistatic models. Under the “Offspring Heterozygous” model of transgenerational epistasis, 72% (29/36) of top association results showed that a model which included both offspring-heterozygous and main effects constituted a significantly better fit than a model including only main effects (LRT *P*-value <0.05). Our most suggestive associations (*P*<10^−5^) which also showed evidence for “Offspring Heterozygous” effect in our multinomial maximum likelihood model (LRT *P*<0.05) were on chromosome 7q in the 3′UTR of *SEMA3C* (rs1527470, *P* = 5.34×10^−6^, LRT *P* = 0.00373), 17q in the intron of *ACCN1* (rs280039, *P* = 7.68×10^−6^, LRT *P* = 4.69×10^−3^) and 18q in the intron of *SLC14A2* (rs959246, *P* = 9.42×10^−6^, LRT *P* = 1.41×10^−4^) (Table 1). All Offspring Heterozygous results with CMH *P*<10^−4^ are listed in Table S6.

We found that in 71% (20/28) of our top maternal-heterozygous effect SNPs, a model which included both maternal-heterozygous and main effects constituted a significantly better fit than a model including only main effects (LRT *P*-value <0.05). Our most suggestive associations (*P*<10^−5^) which also showed evidence for “Maternal Heterozygous” effect in our multinomial maximum likelihood model (LRT *P*<0.05) were on chromosome 9q in the intron of *PALM2* (rs10816846, *P* = 1.44×10^−6^, LRT *P* = 1.39×10^−4^) and on 16p in the intron of *KIAA0430* (rs2071330, *P* = 9.69×10^−6^, LRT *P* = 0.01) (Table 1). All Maternal Heterozygous results with CMH *P*<10^−4^ are listed in Table S7.

We also found that in 78% (38/49) of our top difference effect SNPs a model which included both difference and main effects constituted a significantly better fit than a model including only main effects (LRT *P*-value <0.05). For the “Difference” test, our lowest *P*-values (*P*<10^−5^) which also showed evidence for “Difference” effect in our multinomial maximum likelihood model (likelihood ratio test *P*<0.05) were on chromosomes 4q between *C4orf37* and *UNC5C* (rs28539905, *P* = 1.99×10^−6^, LRT *P* = 0.0206; rs7691268, *P* = 4.12×10^−6^, LRT *P* = 0.0453), on 11p between *INSC* and *SOX6* (rs939046, *P* = 2.03×10^−6^, LRT *P* = 2.46×10^−6^), on 14q in the intron of *SLC7A8* (rs59358210, *P* = 7.86×10^−6^, LRT *P* = 0.00778) and on 15q in the intron of *GABRB3* (rs7171512, *P* = 9.40×10^−6^, LRT *P* = 0.0326) (Table 1). All Difference results with CMH *P*<10^−4^ are listed in Table S8.

Overall, we observed that the MMLM LRT corresponding to the appropriate model provided the best fit for the majority of the top SNPs from each case control test, i.e. for the top offspring-heterozygous effect SNPs a LRT including maternal-heterozygous effects did not produce a greater proportion of significant results than the LRT including the appropriate offspring-heterozygous effect.

### Replication

We attempted replication of results that achieved a significance level of *P*<10^−4^ in our discovery samples using meta-analysis of five imputed family-based autism datasets (N = 3,314 complete affected trios). For the proband main effects, we found a nominal association by TDT on 2q between *NXPH2* and *LRP1B* (rs4245867, discovery *P* = 1.01×10^−5^, replication *P* = 0.024) (Table S4). However, after Bonferroni correction for the number of top results taken into replication, this would not be considered significant.

By comparing maternal and paternal allele frequency (N = 7,019 mothers and fathers) to assess maternal-specific effects, we found a significant replication (meeting a Bonferroni threshold) on 6p in the intron of *CD83* (rs11758033, discovery *P* = 4.48×10^5^, replication *P* = 0.001) (Table S5).

Using maternal vs. paternal genotype mismatch equilibrium tests, we found that no nominal associations were replicated in the “Offspring Heterozygous” or in the “Maternal Heterozygous” model at *P*<0.05 and with an effect size in the same direction as in the discovery samples (Table 1). In the “Difference” model, we found a nominal association in the intron of *TRIM2* (rs10517569, discovery *P* = 6.56×10^−5^, replication *P* = 0.031) (Table S8). This result is not significant considering correction for the number of tests performed in the replication dataset.

## Discussion

Our analysis of proband genetic main effects constitutes a case/control GWAS of autism; previous autism studies employ primarily family-based designs [6], [8], [30], [31], with the exception of one study with both a family-based and case-control subset [7]. A case-control design well-matched for genetic ancestry has some possible advantages compared with family-based studies, including increased power to detect associations at markers with low minor allele frequency, of which we find several in our top results. Using this method we identified associations with SNPs at the *P*<10^−4^ level in or near several genes previously implicated in autism: *NLGN4X*, *RAPGEF4*, *RORA*, *FAM135B* and *CNTNAP2* [34], [35], [36], [37], [38]. One SNP with nominal association in family-based samples is in a region previously reported in a rare deletion in a case with autism features – rs4245867 located between *NXPH2* and *LRP1B* [39]. These findings are promising, but with no results approaching genome-wide significance (*P*<5×10^−8^), they are not definitive. We cannot rule out the possibility that maternal genetic risk factors make no contribution to ASD. Alternatively, our lack of conclusive results may be a direct result of our study size (385 cases and 379 controls); an analysis of power shows that we have 80% power to detect only common variants (>20% minor allele [main effects] or class [TE models] frequency) with genotype relative risk of at least 2 at genome-wide significance, or at least 2.5–3.5 for less common alleles. These effects are larger than those observed for SNP effects in most complex genetic disease, and even in our main effect proband case-control test we did not identify significant findings or replication in family-based data, despite strong evidence that common SNPs contribute to ASD risk [8]. Hence, for a true test of whether or not maternal risk factors contribute to the genetics of ASDs, a more powerful study will be required.

We conducted the first case/control GWAS of maternal genetic main effects which considers having an affected child to be the relevant phenotype. In a recent twin study of autism, Hallmayer *et al*. estimate that the shared environmental component of autism accounts for more of the variability in autism liability than previously appreciated [40]. Given these data, it is important to investigate whether a shared environmental component can be attributed to maternal genetic effects, which could determine the fetal environment. For our analysis of maternal main effects, we anticipated different categories of top results compared with genes represented by proband main effects, for example genes expressed in the placenta as opposed to genes expressed in the brain. The top maternal SNP was in the intron of the gene encoding mastermind-like 2, which is interesting as a candidate in this model because it is a positive regulator of notch signaling and is expressed placentally [41]. Another top signal is near *KLF5*, a transcription factor regulated by mastermind-like signaling and placentally expressed [42]. Surprisingly, we also identified associations with SNPs at the *P*<10^−4^ level near several genes previously implicated in autism, although not in our analysis of proband effects: *PCDH9* and *FOXP1* [43], [44].

Relevant to our models of maternal-fetal interaction, interestingly, one top signal is near a gene known to be immunogenic, *KIAA0430* [45], and one of our significant results in the family-based data is near *CD83*, an immunomodulator also expressed neuronally [46]. The tests of transgenerational epistasis, particularly the “Difference” model, also identified many genes previously implicated in autism at the suggestive *P*<10^−4^ level, such as *MACROD2*, *RORA, SDK1, GABRB3*, *PCDH9, NRXN1*, *RELN*, *FHIT*, *CNTN4* and *LAMA1* [8], [36], [37], [43], [47], [48], [49], [50], [51], [52]. Given the number of reported autism candidate genes, this is not necessarily substantial, however, researchers studying the role of these genes in autism may want to consider examining parental genotype. In support of the transgenerational model, many of these top results were not driven by a proband or maternal main effect. However, no single result reached genome-wide significance or showed strong replication considering the number of tests performed. Many of the reported instances of transgenerational epistasis in animal models have involved a trans-acting effect between independent loci, in addition to a transgenerational effect [53]. In our study we lack sufficient power to search for two-way interactions genome-wide.

We have presented a novel study design and methodology for surveying transgenerational genetic effects on a genome-wide level. By using matched case and control pairs, we can potentially detect over- or under-representation of certain maternal-offspring genotype pairings in autism, and establish the directionality and magnitude of these effects. When we see a putative ‘maternal effect’, we first need to rule out that this result is simply driven by direct risk contributed by the alleles inherited by the proband, which we have done by use of a likelihood ratio test. The second possibility is that there is a main effect of maternal genotype, for example determining the prenatal developmental environment, which we have tested in our maternal main effect model. Finally, we could be observing maternal-fetal genetic interaction, which we have tested in several specific models. We believe that our methods are easy to interpret within this framework on a genome-wide scale and take full advantage of our case/control study design.

One challenge we encountered was the lack of a replication dataset that uses the same study design as our discovery samples. We were able to address this issue by implementing methods that would allow us to detect similar effects using family-based replication datasets. In order to do this, we make the assumption that maternal genetic effects will be distinct from paternal genetic effects, of which there are demonstrated examples [53]. This allows us to use the paternal genotypes and paternal-offspring genotype pairs in our family-based replication as “control” data, while the maternal counterparts are considered to be “case” data. However, this approach would fail to replicate general parental effects that act in both parents, which are also plausible [54].

While we do report nominal associations in our replication data, the number of results which were successfully replicated, as indicated by a *P*-value surviving Bonferroni correction and an effect size in the same direction as in the discovery data, was not more than expected. One factor in this could be the limitations of our imputed replication data. Because the design of the Axiom array is so different from the design of previous arrays, many of the SNPs it assays are poorly covered by the arrays used in our replication datasets. As a result, not all of the top discovery results had equivalent imputed genotype calls with high enough imputation quality to be included in our study, effectively reducing our ability to analyze all markers in our replication data. In our replication method for transgenerational tests this effect is even more exaggerated, since only trios where one parent is heterozygous and one is homozygous are informative. This, combined with data lost during our post-imputation QC, makes our effective replication sample size and marker coverage modest even though we used over 3,000 unique trios for replication.

In conclusion, we have presented a new model for genetic studies of autism that could also be applied to other diseases in order to search for parental genetic effects. Parental main effects of genotype and maternal-fetal incompatibility are important mechanisms to be considered in human disease, and we believe that our methods are a straightforward way to search for these effects on a genome-wide level in case-control or family-based datasets. Although we have identified some promising initial results, individual associations detected here will require replication and further study. Future study of maternal genetic effects could also consider the interaction of maternal genetics with the external environment, for example, maternal exposures during pregnancy.

## Supporting Information

Figure S1 **Multidimensional-scaling plot of the EMA discovery cohort.**
(DOCX)

Figure S2 **Quantile-quantile plots for genome-wide application of our EMA discovery cohort tests.**
(DOCX)

Figure S3 **Quantile-quantile plots for genome-wide application of our transgenerational epistasis replication methods.**
(DOCX)

Methods S1 **Replication Datasets.**
(DOCX)

Methods S2 **Imputation of Replication Datasets.**
(DOCX)

Methods S3 **Stratification of the Discovery Sample.**
(DOCX)

Methods S4 **Multinomial Maximum Likelihood Modeling.**
(DOCX)

Methods S5 **Replication Methods.**
(DOCX)

Table S1 **Replication Datasets.**
(DOCX)

Table S2 **Parameterization of Multinomial Model.**
(DOCX)

Table S3 **Parameters Estimated for the Likelihood Ratio Test.**
(DOCX)

Table S4 **Top results (** ***P*** **<10^−4^) from the CMH test of allele frequencies in the proband samples.**
(DOCX)

Table S5 **Top results (** ***P*** **<10^−4^) from the CMH test of allele frequencies in the maternal samples.**
(DOCX)

Table S6 **Top results (** ***P*** **<10^−4^) using the “Offspring Heterozygous” model of transgenerational epistasis.**
(DOCX)

Table S7 **Top results (** ***P*** **<10^−4^) using the “Maternal Heterozygous” model of transgenerational epistasis.**
(DOCX)

Table S8 **Top results (** ***P*** **<10^−4^) using the “Difference” model of transgenerational epistasis.**
(DOCX)
