# Supplementary material for: A Genome-Wide Survey of Transgenerational Genetic Effects in Autism
Source: PLoS One. 2013 Oct 24;8(10):e76978. doi: 10.1371/journal.pone.0076978 (PMC3811986; doi:10.1371/journal.pone.0076978)
Supplement: Figure S1 — Multidimensional-scaling plot of the EMA discovery cohort. (DOCX) [file pone.0076978.s001.docx]

**Figure_S1:** Multidimensional-scaling plot of the EMA discovery cohort


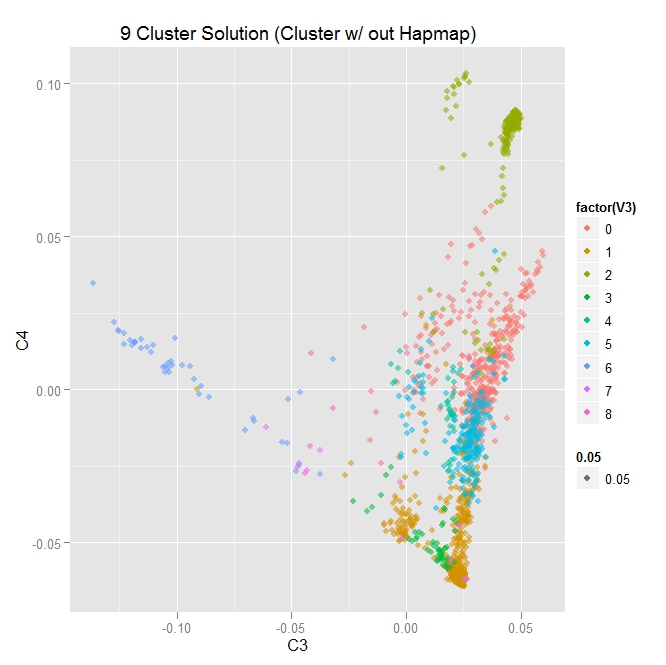


Each point in this plot represents an individual, overall showing the genetic distance between each of our samples and ancestry distribution. Color coded clusters are based on how the samples were divided into a 9 cluster solution implemented in PLINK, 9 clusters being chosen because it minimized genomic inflation. The data for this plot was produced using PLINK’s multidimensional scaling module.
