# Supplementary material for: A Genome-Wide Survey of Transgenerational Genetic Effects in Autism
Source: PLoS One. 2013 Oct 24;8(10):e76978. doi: 10.1371/journal.pone.0076978 (PMC3811986; doi:10.1371/journal.pone.0076978)
Supplement: Figure S2 — Quantile-quantile plots for genome-wide application of our EMA discovery cohort tests. (DOCX) [file pone.0076978.s002.docx]

**Figure_S2**: Quantile-quantile plots for genome-wide application of our EMA discovery cohort tests.


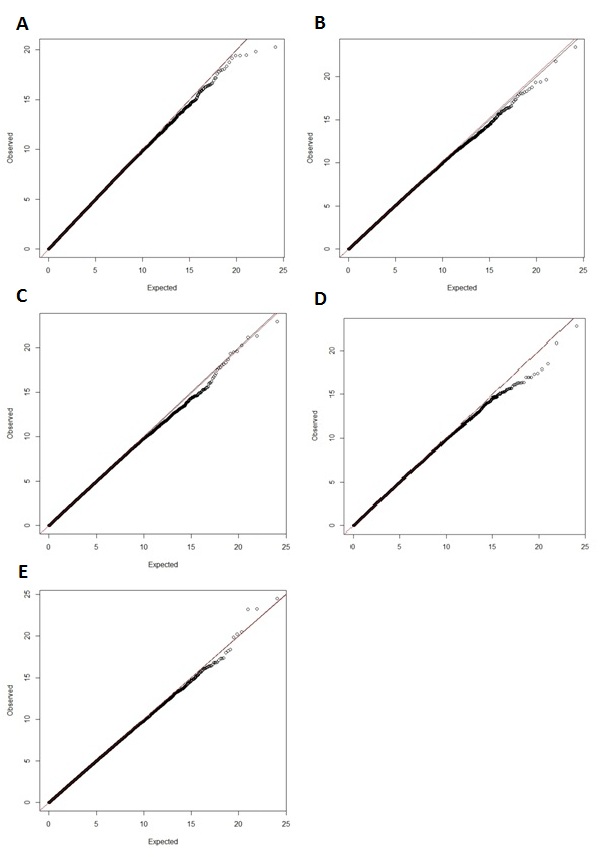


A) Quantile-quantile plot for our CMH test of allele frequency in the EMA offspring (proband main effects), λ = 1.002. B) Quantile-quantile plot for our CMH test of allele frequency in the EMA mothers (maternal main effects), λ = 1.011. C) Quantile-quantile plot for our CMH test of pair-types using the “Offspring Heterozygous” model, λ = 0.991. D) Quantile-quantile plot for our CMH test of pair-types using the “Maternal Heterozygous” model, λ = 0.997. E) Quantile-quantile plot for our CMH test of pair-types using the “Difference” model, λ = 0.998.
