# Supplementary material for: A Genome-Wide Survey of Transgenerational Genetic Effects in Autism
Source: PLoS One. 2013 Oct 24;8(10):e76978. doi: 10.1371/journal.pone.0076978 (PMC3811986; doi:10.1371/journal.pone.0076978)
Supplement: Figure S3 — Quantile-quantile plots for genome-wide application of our transgenerational epistasis replication methods. (DOCX) [file pone.0076978.s003.docx]

**Figure_S3:** Quantile-quantile plots for genome-wide application of our transgenerational epistasis replication methods.


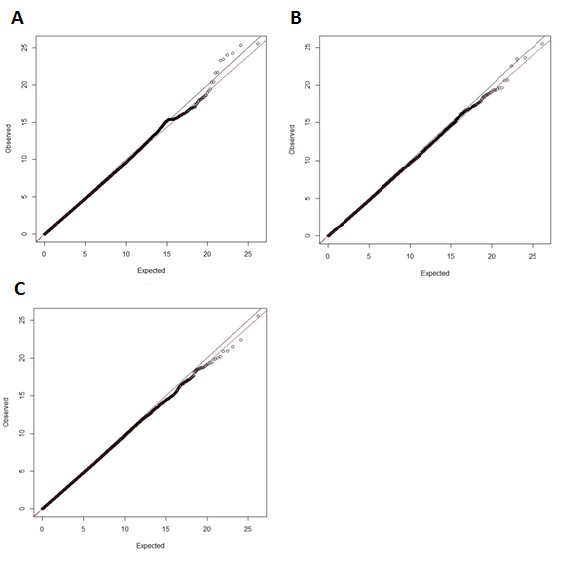


A) Quantile-quantile plot for our “Offspring Heterozygous” replication, λ = 0.995. B) Quantile-quantile plot for our “Maternal Heterozygous” replication, λ = 0.955. C) Quantile-quantile plot

for our “Difference” replication, λ = 0.964.
