# Supplementary material for: A Genome-Wide Survey of Transgenerational Genetic Effects in Autism
Source: PLoS One. 2013 Oct 24;8(10):e76978. doi: 10.1371/journal.pone.0076978 (PMC3811986; doi:10.1371/journal.pone.0076978)
Supplement: Methods S1 — Replication Datasets. (DOCX) [file pone.0076978.s004.docx]

## Method_S1: Replication Datasets

Because these five datasets we used for replication have sample overlap/redundancy, we used PLINK’s IBD module to check for duplicates between all samples. When duplicate samples were uncovered, the individual who was genotyped using an array with lower coverage was discarded. After accounting for sample overlap/redundancy the total size of our replication cohort was 13,373 samples. This included 884 from AGRE/NIMH, 4,074 from AGP, 3,013 from SSC genotyped on the Illumina 1M Duo platform, 1,335 from SSC genotyped on the Illumina 1M platform, and 4,067 from AGRE (Table_S1).
