# Supplementary material for: A Genome-Wide Survey of Transgenerational Genetic Effects in Autism
Source: PLoS One. 2013 Oct 24;8(10):e76978. doi: 10.1371/journal.pone.0076978 (PMC3811986; doi:10.1371/journal.pone.0076978)
Supplement: Methods S2 — Imputation of Replication Datasets. (DOCX) [file pone.0076978.s005.docx]

## Method_S2: Imputation of Replication Datasets

We used the BEAGLE software package in order to do both our phasing and genetic imputation (http://faculty.washington.edu/browning/beagle/beagle.html). We used 1000genomes data for our reference panel available in BEAGLE compatible format by the developers of BEAGLE at (<http://bochet.gcc.biostat.washington.edu/beagle/1000_Genomes.phase1_release_v3/>). The methods for preparing this reference panel are also made available at that location as well. In order to address strand issues between the reference panel and our various datasets we used the strand-switching utility for BEAGLE, which is available at (<http://faculty.washington.edu/sguy/beagle/strand_switching/strand_switching.html>). We used the default settings for all runs of BEAGLE imputation. In order to process our data we divided each dataset into subsets of approximately 300 individuals and 10,000 SNPs each. Each subset had a 1,000 SNP overlap with its neighboring subsets. For each SNP, the imputation resulting from the subset where that SNP was furthest from the edge was used to prevent edge effects in imputation.

Before performing imputation on our replication datasets, we carried out stringent quality control. Because each dataset was collected and genotyped separately, quality control was done separately on each dataset, and different thresholds were used. For AGRE/NIMH-Affymetrix, all markers with HWE *P*-values < 10^-10^ in founder individuals were removed. All markers with a call rate less than 96% were removed. All markers with greater than 10 Mendelian errors were removed. Markers outside of chromosomes 1-22 and X were excluded. In all, 345,066 markers were used for imputation after quality control. Total genomic inflation was calculated to be λ = 1.046.

In the AGP dataset, all markers with HWE *P*-values < 10^-10^ in founder individuals were removed. All markers with a call rate less than 99% were removed. All markers with greater than 10 Mendelian errors were removed. Markers outside of chromosomes 1-22 and X were excluded. In all 749,804 markers were used for imputation after quality control. Total genomic inflation was calculated to be λ = 1.028.

In the AGRE-Illumina dataset, all markers with HWE *P*-values < 10^-3^ in founder individuals were removed. All markers with a call rate less than 95% were removed. All markers with greater than 10 Mendelian errors were removed. Markers outside of chromosomes 1-22 and X were excluded. In all 494,417 markers were used for imputation after quality control. Total genomic inflation was calculated to be λ = 1.055.

For the SSC samples genotyped on the Illumina 1M platform, all markers with HWE *P*-values < 10^-8^ calculated in either founder males or females considered separately were removed. Additionally all markers with HWE *P*-values values < 10^-10^ calculated in males and females founders combined were removed. All markers with a call rate less than 96% were removed. All markers with greater than 10 Mendelian errors were removed. Markers outside of chromosomes 1-22 and X were excluded. In all 1,039,850 markers were used for imputation after quality control. Total genomic inflation was calculated to be λ = 0.9931.

For the SSC samples genotyped on the Illumina 1M Duo platform, all markers with HWE *P*-values < 10^-10^ calculated in either founder males or females considered separately were removed. Additionally, all markers with HWE *P*-values values < 10^-10^ calculated in males and females founders combined were removed. All markers with a call rate less than 96% were removed. All markers with greater than 10 Mendelian errors were removed. In all 1,090,076 markers were used for imputation after quality control. Markers outside of chromosomes 1-22 and X were excluded. Total genomic inflation was calculated to be λ = 1.018.

The resulting imputation data that we used for analysis were the best-guess genotypes provided by BEAGLE. In order to assure high quality we set best-guess genotypes as missing data if they had a posterior probability of less than 0.9. We produced posterior genotype probabilities by using the “gprobs=true” option in BEAGLE.
