# Supplementary material for: A Genome-Wide Survey of Transgenerational Genetic Effects in Autism
Source: PLoS One. 2013 Oct 24;8(10):e76978. doi: 10.1371/journal.pone.0076978 (PMC3811986; doi:10.1371/journal.pone.0076978)
Supplement: Methods S3 — Stratification of the Discovery Sample. (DOCX) [file pone.0076978.s006.docx]

## Method_S3: Stratification of the Discovery Sample

Our discovery cohort was extremely diverse. In order to organize our samples into strata for use in calculating the Cochran-Mantel-Haenzsel statistic we used PLINK’s clustering algorithm. The only clustering restraint we imposed on the algorithm was cluster number (using the --K option). In order to set this tuning parameter in a non-arbitrary fashion, we chose the value of k which minimized the overall genomic inflation in the dataset, as measured by looking at the results of the CMH tests in both the neonates and the mothers. The number of clusters that turned out to minimize the genomic inflation in both tests was 9 (Figure_S1). Using these strata, our genome wide CMH tests in the EMA discovery sample of proband main effects (λ=1.002), maternal main effects (λ=1.011), “Offspring-Heterozygous” (λ=0.991), “Maternal-Heterozygous” (λ=0.997), and “Difference” transgenerational epistatic effects (λ=0.998) had no genomic inflation with accompanying q-q plots shown in Supplementary Figure_S2.
