# Supplementary material for: A Genome-Wide Survey of Transgenerational Genetic Effects in Autism
Source: PLoS One. 2013 Oct 24;8(10):e76978. doi: 10.1371/journal.pone.0076978 (PMC3811986; doi:10.1371/journal.pone.0076978)
Supplement: Methods S4 — Multinomial Maximum Likelihood Modeling. (DOCX) [file pone.0076978.s007.docx]

## Method_S4: Multinomial Maximum Likelihood Modeling

In order to confirm that our maternal main effects were not driven by proband main effects and that putative transgenerational effects were not driven by maternal and/or proband main effects, we carried out a Likelihood Ratio Test (LRT) between models estimating the null parameters with and without the parameter of interest. We fit our models by choosing parameters for estimation using the EMIM software package which is available for download at (http://www.staff.ncl.ac.uk/richard.howey/emim/). Table_S2 describes how all of the different effects we tested fit into a multinomial model of mother-offspring genotype pairs at a single biallelic locus. Table_S3 specifies which parameters were estimated in the null model and in the model of interest for each LRT presented in our results. Note that for our Offspring Heterozygous and Maternal Heterozygous models, there were no corresponding parameter built into EMIM, and as a result we modified the EMIM source code to include these parameters.
