# Supplementary material for: A Genome-Wide Survey of Transgenerational Genetic Effects in Autism
Source: PLoS One. 2013 Oct 24;8(10):e76978. doi: 10.1371/journal.pone.0076978 (PMC3811986; doi:10.1371/journal.pone.0076978)
Supplement: Methods S5 — Replication Methods. (DOCX) [file pone.0076978.s008.docx]

### Method_S5: Replication Methods

We did not apply the Cochran-Mantel-Haenzsel test for our case/control comparison of the mothers and fathers in the replication autism family datasets, since each mother is well matched to a father. In order to demonstrate the soundness of the statistics we used for replication of our transgenerational epistatic effects, we calculated these statistics genome wide in our replication datasets to check for potential genomic inflation. We found that all three statistics to have low genomic inflation, with λ = 0.995 for the “Offspring-Heterozygous” model, λ = 0.955 for the “Maternal-Heterozygous” model, and λ = 0.964 for the “Difference” model (Figure_S3).
