# Supplementary material for: A Genome-Wide Survey of Transgenerational Genetic Effects in Autism
Source: PLoS One. 2013 Oct 24;8(10):e76978. doi: 10.1371/journal.pone.0076978 (PMC3811986; doi:10.1371/journal.pone.0076978)
Supplement: Table S1 — Replication Datasets. (DOCX) [file pone.0076978.s009.docx]

## Table_S1: Replication Datasets

| **Dataset** | **Platform** | **No. Unique Samples** | **Citation** |
| --- | --- | --- | --- |
| AGRE/NIMH | Affymetrix 5.0/500k | 884 | Weiss et al. 2009 [6] |
| AGRE | Illumina 550k | 4,067 | Wang et al. 2009 [7] |
| AGP | Illumina 1M | 4,074 | Anney et al. 2010 [8] |
| SSC | Illumina 1M | 1,335 | Fischbach and Lord 2010 [30]; Sanders et al. 2011 [31] |
|  | Illumina 1M Duo | 3,013 |  |
| **Total** | Imputed (1000genomes reference) | 13,373 | - |

Family-based autism datasets used for replication are shown. For each replication dataset, the name used to refer to the dataset is indicated (Dataset). The platform on which the samples were genotyped is also shown (Platform). In the case of the SSC dataset, the samples were split among two different platforms, the Illumina 1M and 1M Duo. For this reason we considered these to be two different datasets for the purposes of quality control and in our meta-analysis. We list here the number of samples contained in each dataset that are unique among all the datasets (No. Unique Samples). Additionally, we have listed references to papers which have been previously published describing each of these datasets for further reference (Citation).
