# Supplementary material for: A Genome-Wide Survey of Transgenerational Genetic Effects in Autism
Source: PLoS One. 2013 Oct 24;8(10):e76978. doi: 10.1371/journal.pone.0076978 (PMC3811986; doi:10.1371/journal.pone.0076978)
Supplement: Table S2 — Parameterization of Multinomial Model. (DOCX) [file pone.0076978.s010.docx]

**Table_S2: Parameterization of Multinomial Model**

|  | Mother 1/1 | Mother 2/1 | Mother 2/2 |
| --- | --- | --- | --- |
| Child 1/1 | α | α*S_1_*Diff*  Mom-Het | N/A |
| Child 2/1 | α*R_1_*Diff*Off-Het | α*R_1_*S_1_ | α*R_1_*S_2_*Diff*Off-Het |
| Child 2/2 | N/A | α*S_1_*R_2_*Diff*  Mom-Het | α*R_2_*S_2_ |

A summary of the multinomial model that we used to obtain our likelihood ratio test results as implemented in EMIM is shown. Each cell represents one of the 9 possible combinations of maternal and offspring genotype. Two of the cells are marked “N/A” since those combinations are impossible according to Mendelian inheritance. The parameter α represents the baseline probability of having a mother-child genotype pair fall into that cell; in EMIM this baseline probability is estimated from the data. R_1_ and R_2_ represent the coefficients by which this baseline risk is modified when the child possesses one or two copies of the risk allele (in our case, the risk allele is equivalent to the minor allele). S_1_ and S_2_ represent the coefficients by which this baseline risk is modified when the mother possesses one or two copies of the risk allele. “Diff” represents the coefficient by which this baseline risk is modified when the mother and the child do not have identical genotypes. “Mom-Het” represents the coefficient for when the mother is heterozygous and the child is homozygous, and “Off-Het” represents the coefficient for when the mother is homozygous and the child is heterozygous.
