# Supplementary material for: A Genome-Wide Survey of Transgenerational Genetic Effects in Autism
Source: PLoS One. 2013 Oct 24;8(10):e76978. doi: 10.1371/journal.pone.0076978 (PMC3811986; doi:10.1371/journal.pone.0076978)
Supplement: Table S3 — Parameters Estimated for the Likelihood Ratio Test. (DOCX) [file pone.0076978.s011.docx]

**Table_S3: Parameters Estimated for the Likelihood Ratio Test**

| Tested Effect | Null Model | Model of Interest |
| --- | --- | --- |
| Maternal Main | estimate R_1_  R_2_=R_1_² | estimate R_1_  R_2_=R_1_²  estimate S_1_  S_2_=S_1_² |
| Offspring heterozygous | estimate R_1_  R_2_=R_1_²  estimate S_1_  S_2_=S_1_² | estimate R_1_  R_2_=R_1_²  estimate S_1_  S_2_=S_1_²  estimate Offspring heterozygous parameter* |
| Maternal heterozygous | estimate R_1_  R_2_=R_1_²  estimate S_1_  S_2_=S_1_² | estimate R_1_  R_2_=R_1_²  estimate S_1_  S_2_=S_1_²  estimate Maternal heterozygous parameter* |
| Difference | estimate R_1_  R_2_=R_1_²  estimate S_1_  S_2_=S_1_² | estimate R_1_  R_2_=R_1_²  estimate S_1_  S_2_=S_1_²  estimate Li (2009) conflict parameter j_c_§ |

The model options which were specified in EMIM for each implementation of our likelihood ratio test are shown. R_1_ and R_2_ represent the coefficients by which this baseline risk is modified when the child possesses one or two copies of the risk allele (in our case, the risk allele is equivalent to the minor allele). S_1_ and S_2_ represent the coefficients by which this baseline risk is modified when the mother possesses one or two copies of the risk allele. § Corresponds to our Difference model and is an option included with the EMIM package. The first column lists the effect to be tested (Tested Effect). The second column indicates the EMIM options used to specify the null model for each tested effect (Null Model). The third column indicates the EMIM options used to specify the model of interest for each tested effect (Model of Interest). The maximized log likelihoods produced by these models and calculated in EMIM were compared to obtain a LRT *P*-value. Model options marked by an asterisk are not available in the EMIM program by default, and had to be added.
