# Supplementary material for: A Genome-Wide Survey of Transgenerational Genetic Effects in Autism
Source: PLoS One. 2013 Oct 24;8(10):e76978. doi: 10.1371/journal.pone.0076978 (PMC3811986; doi:10.1371/journal.pone.0076978)
Supplement: Table S4 — Top results ( P <10−4) from the CMH test of allele frequencies in the proband samples. (DOCX) [file pone.0076978.s012.docx]

**Table_S4:** Top results (*P* < 10^-4^) from the CMH test of allele frequencies in the proband samples.

| **SNP** | **POS** | **Gene** | **LOC** | **MAF** | **CMH *P*-value** | **OR** | **Prop. of Rep. Data used** | **Rep. *P*-val** | **Rep. OR** |
| --- | --- | --- | --- | --- | --- | --- | --- | --- | --- |
| rs28374251 | 08:005207989 | *CSMD1* | 355661 | 0.39 | 3.48 x 10^-6^ | 0.60 | 0.99 | 0.930 | 1.00 |
| rs7331042 | 13:024783187 | *SPATA13* | intronic | 0.49 | 4.22 x 10^-6^ | 1.64 | Failed to impute | n/a | n/a |
| rs12680005 | 08:139743721 | *COL22A1* | intron | 0.12 | 7.01 x 10^-6^ | 0.48 | Failed to impute | n/a | n/a |
| rs76271340 | 14:055385305 | *GCH1* | 15763 | 0.03 | 9.90 x 10^-6^ | 5.05 | Failed to impute | n/a | n/a |
| rs4245867 | 02:139600715 | *NXPH2* | 62904 | 0.06 | 1.01 x 10^-5^ | 0.36 | 1.00 | 0.024 | 0.88 |
| rs10828479 | 10:018605942 | *CACNB2* | intron | 0.38 | 1.24 x 10^-5^ | 1.63 | 1.00 (SSC1M&Duo) | 0.402 | 0.95 |
| rs79349513 | 07:038603626 | *AMPH* | intron | 0.07 | 1.30 x 10^-5^ | 0.41 | Failed to impute | n/a | n/a |
| rs13031431 | 02:055228835 | *RTN4* | intron | 0.23 | 1.35 x 10^-5^ | 1.72 | 0.96 | 0.315 | 1.07 |
| rs1853155 | 06:123970838 | *TRDN* | 12896 | 0.43 | 1.49 x 10^-5^ | 1.60 | 0.97 | 0.362 | 0.96 |
| rs4659473 | 01:236938621 | *ACTN2* | 11063 | 0.39 | 1.53 x 10^-5^ | 0.63 | 0.94 (AGP, SSC1M&Duo) | 0.596 | 0.97 |
| rs275437 | 05:006879978 | *PAPD7* | 122817 | 0.47 | 2.31 x 10^-5^ | 1.57 | Failed to impute | n/a | n/a |
| rs77842641 | 05:179001459 | *RUFY1* | intron | 0.12 | 2.32 x 10^-5^ | 0.49 | 0.95 | 0.829 | 0.99 |
| rs11176806 | 12:068009946 | *DYRK2* | 32566 | 0.50 | 2.84 x 10^-5^ | 0.64 | Failed to impute | n/a | n/a |
| rs56038012 | 06:051405307 | *PKHD1* | 74838 | 0.02 | 2.87 x 10^-5^ | 5.54 | 1.00 | 0.989 | 1.00 |
| rs7355084 | 01:165567256 | *MGST3* | 32853 | 0.09 | 2.91 x 10^-5^ | 2.15 | 1.00 | 0.705 | 1.02 |
| rs9963570 | 18:070608009 | *NETO1* | 73199 | 0.12 | 3.02 x 10^-5^ | 0.50 | 0.97 | 0.288 | 1.09 |
| rs17430541 | 06:014085980 | *CD83* | 31885 | 0.18 | 3.24 x 10^-5^ | 0.55 | 0.92 | 0.862 | 1.01 |
| rs11726012 | 04:102706018 | *BANK1* | 5746 | 0.32 | 3.65 x 10^-5^ | 0.63 | Failed to impute | n/a | n/a |
| rs3769211 | 02:173914558 | *RAPGEF4* | intron | 0.42 | 4.09 x 10^-5^ | 0.64 | 0.94 | 0.108 | 1.06 |
| rs3808558 | 08:104325059 | *FZD6* | intron | 0.05 | 4.51 x 10^-5^ | 0.36 | 1.00 | 0.382 | 0.94 |
| rs2588519 | 02:055205124 | *RTN4* | intron | 0.33 | 4.64 x 10^-5^ | 1.57 | 0.99 | 0.367 | 1.05 |
| rs12966260 | 18:052898831 | *TCF4* | intron | 0.04 | 5.22 x 10^-5^ | 0.31 | Failed to impute | n/a | n/a |
| rs2787942 | 06:088439383 | *AKIRIN2* | 27398 | 0.12 | 5.65 x 10^-5^ | 0.53 | 0.99 | 0.782 | 0.98 |
| rs4638515 | 15:061733014 | *RORA* | 211512 | 0.19 | 5.85 x 10^-5^ | 1.76 | 0.99 (AGP, SSC1M&Duo) | 0.798 | 1.03 |
| rs7876539 | 23:006198422 | *NLGN4X* | 51716 | 0.22 | 5.94 x 10^-5^ | 1.97 | Failed to impute | n/a | n/a |
| rs73068104 | 03:036334111 | *STAC* | 286986 | 0.32 | 6.72 x 10^-5^ | 1.57 | 0.92 | 0.709 | 1.02 |
| rs78641951 | 09:237398260 | *ELAVL2* | Intron | 0.10 | 6.76 x 10^-5^ | 0.49 | Failed to impute | n/a | n/a |
| rs16908164 | 08:139053333 | *FAM135B* | 88933 | 0.23 | 6.81 x 10^-5^ | 1.69 | 0.93 | 0.327 | 0.94 |
| rs28593037 | 03:035253501 | *ARPP-21* | 430348 | 0.20 | 6.97 x 10^-5^ | 1.71 | 0.96 (SSCDuo Only) | 0.763 | 1.03 |
| rs78951274 | 01:113896333 | *MAGI3* | 37142 | 0.03 | 7.09 x 10^-5^ | 0.23 | Failed to impute | n/a | n/a |
| rs7820729 | 08:132256944 | *ADCY8* | 204109 | 0.47 | 7.27 x 10^-5^ | 0.66 | 0.99 | 0.237 | 0.96 |
| rs1505131 | 18:075860507 | *GALR1* | 850411 | 0.22 | 7.42 x 10^-5^ | 1.64 | 0.99 | 0.068 | 0.91 |
| rs7815115 | 08:005201299 | *CSMD1* | 348971 | 0.34 | 7.71 x 10^-5^ | 0.64 | 0.97 | 0.972 | 1.00 |
| rs3769201 | 02:173956541 | *ZAK* | intron | 0.34 | 7.96 x 10^-5^ | 0.63 | 0.99 | 0.482 | 0.96 |
| rs2109280 | 07:146392067 | *CNTNAP2* | intron | 0.30 | 8.21 x 10^-5^ | 0.62 | 0.97 | 0.854 | 1.01 |
| rs7731657 | 05:129943319 | *CHSY3* | 420992 | 0.33 | 8.25 x 10^-5^ | 0.63 | 0.98 | 0.136 | 1.07 |
| rs6955994 | 07:096307971 | *SHFM1* | 10108 | 0.32 | 8.27 x 10^-5^ | 0.62 | 1.00 | 0.972 | 1.00 |
| rs909927 | 01:169048848 | *ATP1B1* | 27099 | 0.47 | 8.32 x 10^-5^ | 0.65 | 0.95 | 0.432 | 0.97 |
| rs7651759 | 03:110323702 | *PVRL3* | 467163 | 0.33 | 8.34 x 10^-5^ | 1.55 | Failed to impute | n/a | n/a |
| rs73074502 | 07:020624095 | *ABCB5* | 31150 | 0.01 | 8.35 x 10^-5^ | <1 | Failed to impute | n/a | n/a |
| rs56025320 | 17:038756881 | *SMARCE1* | 27099 | 0.03 | 8.37 x 10^-5^ | 3.78 | Failed to impute | n/a | n/a |
| rs13335618 | 16:078804856 | *WWOX* | intron | 0.08 | 8.90 x 10^-5^ | 2.20 | Failed to impute | n/a | n/a |
| rs71356934 | 18:70821796 | *STAC* | 87986 | 0.30 | 8.63 x 10^-5^ | 0.63 | 0.99 | 0.517 | 1.02 |
| rs2327150 | 06:131760040 | *ARG1* | 134325 | 0.03 | 8.86 x 10^-5^ | 3.98 | 1.00 (SSCDuo Only) | 0.401 | 1.17 |

SNPs with *P* < 10^-4^ in the EMA discovery sample test of proband main effects are listed. SNP identity (SNP), chromosome and base-pair position (POS) in hg19 are shown. For each SNP, the closest annotated gene is indicated (Gene), along with the position within the gene or distance from the gene in base-pairs (LOC). Minor allele frequency (MAF) is calculated in the EMA control mothers. Cochran-Mantel-Haenszel (CMH) *P*-values and odds ratios (OR) are shown for the CMH test of allele frequency in EMA autism probands and controls. Replication datasets were imputed to allow maximum coverage of SNPs across different platforms. For each SNP the proportion of samples successfully imputed in our replication dataset is also shown in parentheses (Proportion of Replication Data used). Proportions designating replication datasets in parenthesis indicates that SNP was not imputed across all replication datasets. “Failed to Impute” indicates that imputation failed for that SNP in all replication datasets. Replication *P*-values and odds ratios were calculated by combining the transmission disequilibrium test results from each family-based replication sample using Plink’s random-effects meta-analysis (Rep. *P*-value, Rep. OR). Shaded rows indicate results which had a replication *P-*value < 0.05 and a replication odds ratio in the same direction as in the discovery dataset.
