# Supplementary material for: A Genome-Wide Survey of Transgenerational Genetic Effects in Autism
Source: PLoS One. 2013 Oct 24;8(10):e76978. doi: 10.1371/journal.pone.0076978 (PMC3811986; doi:10.1371/journal.pone.0076978)
Supplement: Table S5 — Top results ( P <10−4) from the CMH test of allele frequencies in the maternal samples. (DOCX) [file pone.0076978.s013.docx]

**Table_S5:** Top results (*P* < 10^-4^) from the CMH test of allele frequencies in the maternal samples.

| **SNP** | **POS** | **Gene** | **LOC** | **MAF** | **CMH *P*-value** | **OR** | **EMIM LRT *P*-value** | **Prop. of Rep. Data used** | **Rep. *P*-val** | **Rep. OR** |
| --- | --- | --- | --- | --- | --- | --- | --- | --- | --- | --- |
| rs1940153 | 11:095789959 | *MAML2* | intron | 0.43 | 2.05 x 10^-6^ | 1.68 | 1.22 x 10^-3^ | 0.93 | 0.542 | 1.02 |
| rs9895531 | 17:010050671 | *GAS7* | intron | 0.48 | 5.45 x 10^-6^ | 1.61 | 4.79 x 10^-6^ | 0.93 | 0.540 | 0.98 |
| rs7528615 | 01:084732963 | *PRKACB* | 28782 | 0.35 | 6.19 x 10^-6^ | 0.61 | 9.63 x 10^-5^ | 1.00 | 0.548 | 0.99 |
| rs2006933 | 17:026601133 | *TMEM97* | 44988 | 0.26 | 7.81 x 10^-6^ | 1.71 | 3.59 x 10^-5^ | 0.96 | 0.099 | 1.07 |
| rs8001767 | 13:073816825 | *KLF5* | 165149 | 0.27 | 9.95 x 10^-6^ | 1.72 | 8.24 x 10^-4^ | 0.97 | 0.218 | 0.95 |
| rs1814474 | 10:049797216 | *ARHGAP22* | intron | 0.18 | 1.36 x 10^-5^ | 1.83 | 1.29 x 10^-4^ | 0.99 | 0.072 | 0.91 |
| rs73431944 | 11:023624266 | *SVIP* | 772884 | 0.08 | 1.54 x 10^-5^ | 2.39 | 5.43 x 10^-5^ | 0.93 | 0.599 | 0.97 |
| rs76282240 | 03:112490664 | *CD200R1L* | 43892 | 0.04 | 1.56 x 10^-5^ | 0.28 | 5.37 x 10^-4^ | failed to impute | n/a | n/a |
| rs12988126 | 02:049246729 | *FSHR* | intron | 0.33 | 1.83 x 10^-5^ | 1.61 | 2.71 x 10^-4^ | 0.91 | 0.383 | 1.03 |
| rs6133869 | 20:010320196 | *SNAP25* | 32131 | 0.42 | 2.12 x 10^-5^ | 1.56 | 3.28 x 10^-5^ | 1.00 | 0.967 | 1.00 |
| rs9287655 | 02:015385484 | *NBAS* | intron | 0.41 | 2.25 x 10^-5^ | 0.63 | 3.61 x 10^-2^ | 0.97 | 0.587 | 0.98 |
| rs11612103 | 12:030193389 | *TMTC1* | 255697 | 0.19 | 2.30 x 10^-5^ | 1.75 | 2.64 x 10^-4^ | 0.96 | 0.463 | 1.03 |
| rs11636863 | 15:036347729 | *ATPBD4* | 509325 | 0.18 | 2.69 x 10^-5^ | 1.80 | 9.33 x 10^-4^ | failed to impute | n/a | n/a |
| rs11615322 | 12:030195415 | *TMTC1* | 257723 | 0.05 | 2.81 x 10^-5^ | 2.74 | 7.29 x 10^-4^ | 0.97 | 0.367 | 0.93 |
| rs16963439 | 15:050516707 | *SLC27A2* | intron | 0.15 | 2.83 x 10^-5^ | 1.84 | 4.06 x 10^-6^ | 1.00 | 0.993 | 1.00 |
| rs2288273 | 03:101378927 | *ZBTB11* | intron | 0.18 | 3.00 x 10^-5^ | 0.55 | 1.23 x 10^-4^ | 0.97 | 0.364 | 1.06 |
| rs293090 | 05:004563324 | *LOC340094* | 471148 | 0.25 | 3.02 x 10^-5^ | 1.67 | 5.87 x 10^-3^ | 0.95 (AGP, AGRE, SSC1M&Duo) | 0.576 | 0.98 |
| rs935291 | 10:049786443 | *ARHGAP22* | intron | 0.16 | 3.07x 10^-5^ | 1.82 | 2.44 x 10^-4^ | 1.00 | 0.014 | 0.89 |
| rs61374830 | 09:014030190 | *NFIB* | 51657 | 0.12 | 4.26 x 10^-5^ | 1.99 | 1.63 x 10^-3^ | failed to impute | n/a | n/a |
| rs11758033 | 06:014129616 | *CD83* | intron | 0.16 | 4.48 x 10^-5^ | 0.55 | 7.36 x 10^-5^ | 1.00 | 0.001 | 0.86 |
| rs2038912 | 10:020207349 | *PLXDC2* | intron | 0.49 | 4.57 x 10^-5^ | 1.53 | 7.70 x 10^-6^ | 0.97 | 0.481 | 1.04 |
| rs77858930 | 02:004013571 | *ALLC* | 263311 | 0.08 | 4.59 x 10^-5^ | 2.20 | 8.18 x 10^-4^ | 1.00 (SSCDuo only) | 0.934 | 0.99 |
| rs1882558 | 02:049247737 | *FSHR* | intron | 0.29 | 4.64 x 10^-5^ | 1.60 | 9.35 x 10^-5^ | 0.91 | 0.432 | 1.03 |
| rs189927265 | 15:082532510 | *EFTUD1* | intron | 0.02 | 4.74 x 10^-5^ | 6.70 | 1.65 x 10^-4^ | 1.00 | 0.172 | 0.85 |
| rs11087010 | 20:010329195 | *SNAP25* | 41130 | 0.20 | 4.83 x 10^-5^ | 1.68 | 2.58 x 10^-4^ | 1.00 | 0.280 | 1.05 |
| rs35184915 | 23:037605765 | *XK* | 14382 | 0.05 | 5.03 x 10^-5^ | 2.74 | n/a | n/a | n/a | n/a |
| rs7987479 | 13:092149642 | *GPC5* | intron | 0.03 | 5.27 x 10^-5^ | 4.64 | 1.43 x 10^-3^ | failed to impute | n/a | n/a |
| rs1327449 | 01:071337925 | *PTGER3* | intron | 0.10 | 5.29 x 10^-5^ | 0.49 | 4.17 x 10^-4^ | 0.99 | 0.179 | 1.06 |
| rs9592356 | 13:065199850 | *PCDH9* | 1677117 | 0.34 | 5.30 x 10^-5^ | 0.64 | 4.36 x 10^-4^ | 0.90 | 0.498 | 0.99 |
| rs80122763 | 08:027083124 | *STMN4* | 10690 | 0.07 | 5.56 x 10^-5^ | 2.31 | 6.17 x 10^-5^ | failed to impute | n/a | n/a |
| rs7540329 | 01:247486678 | *ZNF496* | intron | 0.45 | 5.75 x 10^-5^ | 0.66 | 2.27 x 10^-4^ | 0.99 | 0.673 | 0.99 |
| rs11830731 | 12:009803933 | *LOC374443* | intron | 0.03 | 5.78 x 10^-5^ | 3.54 | 6.71 x 10^-3^ | 0.97 | 0.642 | 0.96 |
| rs2171800 | 02:066461479 | *MEIS1* | 201053 | 0.33 | 5.89 x 10^-5^ | 1.55 | 8.81 x 10^-3^ | 0.96 | 0.583 | 1.02 |
| rs56260555 | 13:029973061 | *KIAA0774* | intron | 0.30 | 5.96 x 10^-5^ | 0.63 | 5.33 x 10^-4^ | failed to impute | n/a | n/a |
| rs73113731 | 03:007384357 | *GRM7* | intron | 0.01 | 6.79 x 10^-5^ | < 1.0 | 1.92 x 10^-4^ | 0.99 | 0.974 | 0.99 |
| rs72737059 | 15:050502084 | *SLC27A2* | intron | 0.10 | 6.82 x 10^-5^ | 1.98 | 6.53 x 10^-5^ | 0.97 | 0.577 | 0.95 |
| rs73431912 | 11:023594544 | *SVIP* | 743162 | 0.07 | 6.86 x 10^-5^ | 2.38 | 5.39 x 10^-4^ | failed to impute | n/a | n/a |
| rs79053063 | 12:112550965 | *NAA25* | 86472 | 0.04 | 6.87 x 10^-5^ | 3.19 | 6.41 x 10^-3^ | 0.95 | 0.539 | 0.96 |
| rs73466557 | 06:082003889 | *FAM46A* | 451558 | 0.04 | 7.06 x 10^-5^ | 3.44 | 4.01 x 10^-3^ | 0.99 | 0.094 | 1.13 |
| rs2209169 | 01:178601492 | *RALGPS2* | 92808 | 0.48 | 7.33 x 10^-5^ | 0.66 | 1.10 x 10^-2^ | 0.99 | 0.683 | 0.98 |
| rs7226182 | 17:026653207 | *TMEM97* | intron | 0.12 | 7.41 x 10^-5^ | 1.94 | 1.65 x 10^-4^ | 1.00 | 0.830 | 0.96 |
| rs17022431 | 04:147977816 | *TTC29* | 110782 | 0.03 | 7.79 x 10^-5^ | 0.25 | 1.12 x 10^-4^ | 0.98 | 0.716 | 1.03 |
| rs12604950 | 18:074036299 | *ZNF516* | 33345 | 0.06 | 7.80 x 10^-5^ | 2.46 | 5.35 x 10^-2^ | 0.92 (AGRE, SSC1M&Duo) | 0.918 | 0.98 |
| rs9918478 | 06:093718878 | *EPHA7* | 230860 | 0.03 | 7.81 x 10^-5^ | 3.52 | 9.59 x 10^-2^ | 0.98 | 0.515 | 0.94 |
| rs2377517 | 10:049793515 | *ARHGAP22* | intron | 0.13 | 7.83 x 10^-5^ | 1.88 | 2.60 x 10^-4^ | 1.00 | 0.041 | 0.88 |
| rs75114276 | 03:070631640 | *FOXP1* | 373096 | 0.04 | 7.92 x 10^-5^ | 2.85 | 1.05 x 10^-3^ | failed to impute | n/a | n/a |
| rs72970947 | 11:093306988 | *C11orf75* | 30442 | 0.05 | 8.06 x 10^-5^ | 2.62 | 3.32 x 10^-3^ | 0.99 | 0.228 | 1.08 |
| rs35340808 | 04:014784441 | *CPEB2* | 221081 | 0.19 | 8.13 x 10^-5^ | 1.69 | 2.13 x 10^-3^ | 0.94 | 0.511 | 1.06 |
| rs1858545 | 01:099354511 | *LPPR5* | 1289 | 0.02 | 8.20 x 10^-5^ | 5.57 | 4.78 x 10^-2^ | failed to impute | n/a | n/a |
| rs75879230 | 01:086069654 | *CYR61* | 20008 | 0.01 | 8.25 x 10^-5^ | 0.05 | 6.15 x 10^-4^ | 0.96 | 0.748 | 0.75 |
| rs12604929 | 18:020824373 | *CABLES1* | intron | 0.41 | 8.41 x 10^-5^ | 1.51 | 1.69 x 10^-3^ | 0.94 | 0.269 | 0.96 |
| rs55642547 | 17:045931325 | *SP6* | intron | 0.25 | 8.48 x 10^-5^ | 1.64 | 3.02 x 10^-2^ | 0.97 | 0.446 | 1.03 |
| rs5989534 | 23:006591329 | *VCX3A* | 138170 | 0.27 | 8.58 x 10^-5^ | 1.58 | n/a | n/a | n/a | n/a |
| rs78622085- | 13:021866587 | *ZDHHC20* | 83921 | 0.06 | 9.29 x 10^-5^ | 2.49 | 1.60 x 10^-2^ | 0.91 (AGP, AGRE, SSCDuo) | 0.985 | 1.00 |
| rs7996589 | 13:073815899 | *KLF5* | 164223 | 0.41 | 9.34 x 10^-5^ | 1.51 | 1.60 x 10^-3^ | 0.94 | 0.180 | 1.05 |
| rs7174016 | 15:098526851 | *ARRDC4* | 9783 | 0.06 | 9.68 x 10^-5^ | 2.60 | 5.97 x 10^-6^ | 0.93 (AGP, SSC1M&Duo) | 0.028 | 0.72 |
| rs7173583 | 15:098517649 | *ARRDC4* | 581 | 0.05 | 9.69 x 10^-5^ | 2.70 | 1.57 x 10^-5^ | 0.93 (AGP, SSC1M&Duo) | 0.080 | 0.76 |
| rs74705317 | 11:108659417 | *DDX10* | intron | 0.01 | 9.84 x 10^-5^ | > 1.0 | 2.61 x 10^-3^ | 0.93 | 0.440 | 1.29 |

SNPs with *P* < 10^-4^ in the EMA discovery sample test of maternal main effects are listed. SNP identity (SNP), chromosome and base-pair position (POS) in hg19 are shown. For each SNP, the closest annotated gene is indicated (Gene), along with the position within the gene or distance from the gene in base-pairs (LOC). Minor allele frequency (MAF) is calculated in the EMA control mothers. *P*-values (CMH *P*-value) and odds ratios (OR) are shown for the Cochran-Mantel-Haenszel (CMH) test of allele frequency in EMA case and control mothers. In order to show that our maternal main effects are not driven by proband main effects, a comparison between multinomial models including both maternal and proband main and only proband main effects is shown (LRT *P*-value of Maternal Effect); this analysis was not possible for SNPs on sex chromosomes as indicated by “N/A”. Replication datasets were imputed to allow maximum coverage of SNPs across different platforms. For each SNP the proportion of samples successfully imputed in our replication dataset is also shown in parentheses (Proportion of Replication Data used). Proportions designating replication datasets in parenthesis indicates that SNP was not imputed across all replication datasets. Replication *P*-values and odds ratios were calculated by carrying out a chi-square test of allele frequencies in family based replication datasets, considering mothers as “affected” and fathers as “unaffected”; results were then combined across separate replication datasets using Plink’s random-effects meta-analysis (Rep. *P*-value, Rep. OR). “Failed to Impute” indicates that imputation failed for that SNP in all replication datasets. Shaded rows indicate results which had a replication *P-*value < 0.05 and a replication odds ratio in the same direction as in the discovery dataset.
