# Supplementary material for: A Genome-Wide Survey of Transgenerational Genetic Effects in Autism
Source: PLoS One. 2013 Oct 24;8(10):e76978. doi: 10.1371/journal.pone.0076978 (PMC3811986; doi:10.1371/journal.pone.0076978)
Supplement: Table S8 — Top results ( P <10−4) using the “Difference” model of transgenerational epistasis. (DOCX) [file pone.0076978.s016.docx]

**Table_S8**: Top results (*P* < 10^-4^) using the “Difference” model of transgenerational epistasis.

| **SNP** | **POS** | **Gene** | **LOC** | **MAF** | **CMH *P*-value** | **OR** | **C/C proband *P*-Value** | **C/C proband**  **OR** | **C/C Maternal *P*-Value** | **C/C Maternal OR** | **LRT *P*-value of Difference** | **Prop. of Rep. Data used** | **Rep. *P*-val** | **Rep. OR** |
| --- | --- | --- | --- | --- | --- | --- | --- | --- | --- | --- | --- | --- | --- | --- |
| rs28539905 | 04:098241477 | *C4orf37* | 238550 | 0.11 | 1.99 x 10^-6^ | 2.57 | 2.56 x 10^-2^ | 1.44 | 9.23 x 10^-3^ | 1.53 | 2.06 x 10^-2^ | 0.99 | 0.153 | 1.11 |
| rs939046 | 11:015275242 | *INSC* | 6488 | 0.39 | 2.03 x 10^-6^ | 0.48 | 7.11 x 10^-1^ | 0.96 | 8.46 x 10^-1^ | 1.02 | 2.46 x 10^-6^ | 0.93 | 0.025 | 1.10 |
| rs7691268 | 04:098233960 | *C4orf37* | 246067 | 0.10 | 4.12 x 10^-6^ | 2.61 | 1.83 x 10^-2^ | 1.50 | 3.95 x 10^-3^ | 1.64 | 4.53 x 10^-2^ | 1.00 | 0.074 | 1.17 |
| rs59358210 | 14:023631372 | *SLC7A8* | intronic | 0.10 | 7.86 x 10^-6^ | 0.41 | 2.45 x 10^-1^ | 0.81 | 3.81 x 10^-3^ | 0.61 | 7.78 x 10^-3^ | failed to impute | n/a | n/a |
| rs7171512 | 15:026906345 | *GABRB3* | intronic | 0.22 | 9.40 x 10^-6^ | 2.12 | 2.28 x 10^-1^ | 1.17 | 1.82 x 10^-1^ | 1.19 | 3.26 x 10^-2^ | 1.0 (AGRE/NIMH only) | 0.185 | 0.82 |
| rs34106291 | 03:031851186 | *OSBPL10* | intronic | 0.06 | 1.40 x 10^-5^ | 3.02 | 3.60 x 10^-1^ | 1.21 | 1.25 x 10^-1^ | 1.38 | 3.95 x 10^-2^ | failed to impute | n/a | n/a |
| rs73007340 | 11:111090479 | *C11orf53* | 36228 | 0.06 | 1.55 x 10^-5^ | 0.32 | 1.36 x 10^-2^ | 0.56 | 9.32 x 10^-2^ | 0.68 | 6.87 x 10^-4^ | failed to impute | n/a | n/a |
| rs113252007 | 20:015563026 | *MACROD2* | intronic | 0.04 | 1.58 x 10^-5^ | 0.27 | 4.35 x 10^-1^ | 0.81 | 5.72 x 10^-1^ | 0.86 | 6.97 x 10^-3^ | failed to impute | n/a | n/a |
| rs1865971 | 16:059031168 | *GOT2* | 262922 | 0.49 | 1.91 x 10^-5^ | 1.93 | 7.46 x 10^-1^ | 1.04 | 4.72 x 10^-1^ | 0.92 | 6.92 x 10^-1^ | failed to impute | n/a | n/a |
| rs75270576 | 03:005779007 | *EDEM1* | 517358 | 0.02 | 2.31 x 10^-5^ | 0.14 | 7.68 x 10^-4^ | 0.29 | 1.08 x 10^-3^ | 0.19 | 4.00 x 10^-5^ | failed to impute | n/a | n/a |
| rs35550910 | 02:052228977 | *NRXN1* | 969303 | 0.04 | 2.69 x 10^-5^ | 4.06 | 2.02 x 10^-1^ | 1.44 | 8.85 x 10^-2^ | 1.61 | 5.45 x 10^-4^ | 0.97 | 0.254 | 1.14 |
| rs7510120 | 21:022432784 | *NCAM2* | intronic | 0.05 | 2.74 x 10^-5^ | 0.34 | 2.74 x 10^-2^ | 0.58 | 4.78 x 10^-3^ | 0.51 | 1.94 x 10^-3^ | 0.96 | 0.520 | 1.05 |
| rs1672879 | 12:125281102 | *SCARB1* | intronic | 0.24 | 2.80 x 10^-5^ | 2.13 | 4.25 x 10^-1^ | 0.90 | 6.53 x 10^-1^ | 0.94 | 4.91 x 10^-1^ | 0.91 | 0.851 | 1.02 |
| rs10900954 | 06:000483817 | *EXOC2* | 1321 | 0.37 | 3.03 x 10^-5^ | 1.89 | 4.10 x 10^-1^ | 1.09 | 1.34 x 10^-1^ | 1.18 | 9.02 x 10^-3^ | 0.99 | 0.821 | 1.01 |
| rs55870187 | 07:077620868 | *MAGI2* | 25506 | 0.30 | 3.07 x 10^-5^ | 1.94 | 5.91 x 10^-1^ | 1.07 | 8.63 x 10^-2^ | 1.22 | 5.46 x 10^-2^ | failed to impute | n/a | n/a |
| rs1835112 | 05:081407916 | *ATG10* | intronic | 0.29 | 3.24 x 10^-5^ | 0.52 | 4.27 x 10^-1^ | 0.91 | 7.73 x 10^-2^ | 0.82 | 1.38 x 10^-3^ | 0.95 | 0.830 | 1.01 |
| rs4141264 | 20:052557187 | *BCAS1* | 2892 | 0.38 | 3.30 x 10^-5^ | 0.53 | 4.78 x 10^-3^ | 0.74 | 1.18 x 10^-2^ | 0.76 | 1.95 x 10^-7^ | failed to impute | n/a | n/a |
| rs78562699 | 04:183814089 | *DCTD* | intronic | 0.01 | 3.35 x 10^-5^ | 20.41 | 9.61 x 10^-2^ | 2.37 | 1.46 x 10^-3^ | 5.34 | 3.16 x 10^-3^ | 0.91 (SSCDuo only) | 0.273 | 1.62 |
| rs959246 | 18:043176336 | *SLC14A2* | 18430 | 0.25 | 3.36 x 10^-5^ | 0.52 | 1.10 x 10^-2^ | 0.74 | 9.24 x 10^-2^ | 0.82 | 1.31 x 10^-3^ | 0.95 | 0.694 | 1.02 |
| rs74367993 | 01:226602765 | *PARP1* | 6964 | 0.03 | 3.41 x 10^-5^ | 0.16 | 4.85 x 10^-1^ | 1.26 | 5.23 x 10^-1^ | 1.22 | 3.28 x 10^-6^ | failed to impute | n/a | n/a |
| rs7242969 | 18:069620818 | *CBLN2* | 583097 | 0.10 | 4.30 x 10^-5^ | 2.29 | 6.16 x 10^-3^ | 1.61 | 2.57 x 10^-2^ | 1.48 | 1.06 x 10^-3^ | 1.00 (SSCDuo only) | 0.817 | 1.03 |
| rs11207552 | 01:060439833 | *C1orf87* | 16233 | 0.47 | 4.68 x 10^-5^ | 1.85 | 4.75 x 10^-1^ | 1.08 | 3.43 x 10^-1^ | 0.91 | 4.33 x 10^-2^ | failed to impute | n/a | n/a |
| rs12746334 | 01:235708538 | *GNG4* | 2447 | 0.09 | 4.77 x 10^-5^ | 2.41 | 4.23 x 10^-1^ | 1.15 | 6.45 x 10^-1^ | 1.09 | 3.45 x 10^-2^ | 1.00 | 0.514 | 1.00 |
| rs1716451 | 12:119981973 | *CCDC60* | 3122 | 0.13 | 4.80 x 10^-5^ | 2.15 | 2.93 x 10^-3^ | 1.56 | 2.03 x 10^-2^ | 1.42 | 1.86 x 10^-3^ | 1.00 | 0.904 | 1.01 |
| rs10514196 | 18:073680098 | *ZNF516* | 389546 | 0.10 | 4.85 x 10^-5^ | 0.45 | 2..35 x 10^-4^ | 0.54 | 2.85 x 10^-2^ | 0.69 | 4.89 x 10^-5^ | 0.98 | 0.850 | 1.01 |
| rs73179537 | 08:002761962 | *CSMD1* | 30914 | 0.11 | 5.00 x 10^-5^ | 0.43 | 2.71 x 10^-1^ | 0.83 | 2.17 x 10^-1^ | 0.81 | 5.43 x 10^-3^ | 0.97 | 0.765 | 1.02 |
| rs5939125 | 23:002733210 | *XG* | intronic | 0.37 | 5.53 x 10^-5^ | 1.87 | 4.11 x 10^-2^ | 1.36 | 1.73 x 10^-1^ | 1.16 | n/a | n/a | n/a | n/a |
| rs11374741 | 21:027261091 | *APP* | intronic | 0.42 | 5.76 x 10^-5^ | 1.86 | 6.77 x 10^-1^ | 0.96 | 8.48 x 10^-1^ | 1.02 | 2.26 x 10^-3^ | Failed to Impute | n/a | n/a |
| rs12503529 | 04:023873828 | *PPARGC1A* | intronic | 0.13 | 5.85 x 10^-5^ | 2.11 | 3.74 x 10^-1^ | 1.15 | 3.29 x 10^-1^ | 1.16 | 1.78 x 10^-2^ | failed to impute | n/a | n/a |
| rs10032416 | 04:068704829 | *TMPRSS11D* | intronic | 0.26 | 5.86 x 10^-5^ | 0.53 | 2.61 x 10^-2^ | 0.77 | 7.47 x 10^-2^ | 0.81 | 3.30 x 10^-3^ | failed to impute | n/a | n/a |
| rs1330910 | 13:078582044 | *EDNRB* | 32380 | 0.07 | 5.92 x 10^-5^ | 0.38 | 2.12 x 10^-1^ | 0.77 | 1.09 x 10^-1^ | 0.71 | 2.47 x 10^-3^ | 1.00 | 0.686 | 1.03 |
| rs160414 | 08:090637625 | *RIPK2* | 132350 | 0.25 | 5.92 x 10^-5^ | 1.90 | 5.13 x 10^-2^ | 1.26 | 3.59 x 10^-1^ | 1.12 | 8.32 x 10^-3^ | 0.94 | 0.040 | 1.10 |
| rs7628934 | 03:087175984 | *CHMP2B* | 100429 | 0.39 | 6.38 x 10^-5^ | 1.84 | 9.41 x 10^-1^ | 0.99 | 2.81 x 10^-1^ | 1.12 | 6.79 x 10^-2^ | 0.97 | 0.893 | 1.01 |
| rs78270488 | 04:112438016 | *C4orf32* | 628537 | 0.03 | 6.54 x 10^-5^ | 0.20 | 3.62 x 10^-2^ | 0.48 | 1.32 x 10^-3^ | 0.34 | 2.25 x 10^-3^ | 0.92 | 0.585 | 1.06 |
| rs10517569 | 04:154244953 | *TRIM2* | intronic | 0.12 | 6.56 x 10^-5^ | 2.19 | 5.35 x 10^-4^ | 1.76 | 2.30 x 10^-1^ | 1.21 | 4.65 x 10^-3^ | 1.00 | 0.031 | 1.12 |
| rs10213433 | 04:098208093 | *C4orf37* | 271934 | 0.11 | 6.58 x 10^-5^ | 2.19 | 1.98 x 10^-2^ | 1.47 | 1.44 x 10^-2^ | 1.50 | 7.49 x 10^-2^ | 0.97 | 0.098 | 1.13 |
| rs4444152 | 12:075257177 | *KCNC2* | 176719 | 0.45 | 6.67 x 10^-5^ | 1.84 | 5.75 x 10^-1^ | 0.94 | 1.28 x 10^-1^ | 0.85 | 1.52 x 10^-1^ | 0.95 | 0.751 | 0.99 |
| rs17181170 | 03:087173324 | *CHMP2B* | 103089 | 0.40 | 7.57 x 10^-5^ | 1.84 | 8.18 x 10^-1^ | 0.98 | 2.84 x 10^-1^ | 1.12 | 6.24 x 10^-2^ | 0.98 | 0.969 | 1.00 |
| rs1202232 | 07:003647891 | *SDK1* | intronic | 0.39 | 7.74 x 10^-5^ | 1.82 | 5.93 x 10^-1^ | 1.06 | 5.10 x 10^-1^ | 1.07 | 7.85 x 10^-2^ | 0.98 | 0.498 | 0.97 |
| rs11805261 | 01:168095261 | *GPR161* | intronic | 0.10 | 8.21 x 10^-5^ | 0.46 | 3.35 x 10^-2^ | 0.68 | 8.53 x 10^-3^ | 0.63 | 2.55 x 10^-2^ | 1.00 | 0.367 | 1.06 |
| rs80254797 | 04:141011869 | *MAML3* | intronic | 0.02 | 8.39 x 10^-5^ | 5.65 | 9.95 x 10^-2^ | 2.01 | 5.43 x 10^-2^ | 2.02 | 1.21 x 10^-3^ | Failed to Impute | n/a | n/a |
| rs28370576 | 18:025934563 | *CDH2* | 177118 | 0.31 | 8.51 x 10^-5^ | 1.84 | 8.71 x 10^-1^ | 1.02 | 6.65 x 10^-1^ | 0.95 | 5.51 x 10^-1^ | failed to impute | n/a | n/a |
| rs722277 | 07:103595652 | *RELN* | intronic | 0.35 | 8.69 x 10^-5^ | 1.82 | 3.56 x 10^-1^ | 1.11 | 2.13 x 10^-1^ | 1.15 | 5.26 x 10^-3^ | 0.97 | 0.549 | 1.02 |
| rs6824610 | 04:076781677 | *PPEF2* | 3UTR | 0.01 | 8.73 x 10^-5^ | 0.04 | 1.75 x 10^-2^ | 0.36 | 1.76 x 10^-1^ | 0.52 | 2.70 x 10^-3^ | 0.98 | 0.244 | 0.73 |
| rs67735550 | 02:198229453 | *SF3B1* | 27245 | 0.36 | 8.75 x 10^-5^ | 1.82 | 2.23 x 10^-1^ | 1.15 | 1.20 x 10^-1^ | 1.18 | 6.42 x 10^-3^ | 0.91 (SSC1M&Duo) | 0.785 | 0.98 |
| rs9436636 | 01:061812773 | *NFIA* | intronic | 0.33 | 8.82 x 10^-5^ | 0.55 | 5.47 x 10^-1^ | 0.94 | 1.87 x 10^-1^ | 0.86 | 6.05 x 10^-3^ | 0.99 (AGRE/NIMH, SSC1M&Duo) | 0.415 | 0.94 |
| rs1918482 | 07:094843104 | *PPP1R9A* | intronic | 0.48 | 9.09 x 10^-5^ | 1.81 | 9.81 x 10^-1^ | 1.00 | 8.06 x 10^-1^ | 0.98 | 1.64 x 10^-3^ | 0.98 | 0.992 | 1.00 |
| rs6507625 | 18:043186842 | *SLC14A2* | intronic | 0.26 | 9.51 x 10^-5^ | 0.55 | 1.40 x 10^-2^ | 0.75 | 4.37 x 10^-2^ | 0.79 | 1.35 x 10^-3^ | 0.99 | 0.517 | 1.04 |

SNPs with *P* < 10^-4^ in the EMA discovery sample test of transgenerational epistasis using the “Difference” model are listed. SNP identity (SNP), chromosome and base-pair position (POS) in hg19 are shown. For each SNP, the closest annotated gene is indicated (Gene), along with the position within the gene or distance from the gene in base-pairs (LOC). Minor allele frequency (MAF) is calculated in the EMA control mothers. *P*-values (CMH *P*-value) and odds ratios (OR) are shown for a Cochran-Mantel-Haenszel (CMH) test of pair-type counts in case vs. control pairs from the EMA discovery cohort. Additionally, CMH *P*-values and odds ratios for proband and maternal main effects as calculated in the EMA discovery cohort are shown for comparison (C/C proband *P*, C/C proband OR, C/C maternal *P*, C/C maternal OR). In order to show that our difference transgenerational epistatic effects are not driven by proband and maternal main effects, a comparison between multinomial models including both difference effects and main effects and only main effects is shown (LRT *P*-value of Difference). Replication datasets were imputed to allow maximum coverage of SNPs across different platforms. For each SNP the proportion of samples successfully imputed in our replication dataset is also shown in parentheses (Prop. of Rep. Data used). Proportions designating replication datasets in parenthesis indicates that SNP was not imputed across all replication datasets. Replication was performed on trios with one homozygous and one heterozygous parent by chi-square test of whether the mother is disproportionately the parent with a non-identical genotype compared to the proband; results were then combined across replication datasets using Plink’s random-effects meta-analysis (Rep. *P*-value, Rep. OR).
